# Loss of *GPRC5D* enhances the proliferative capacity and competitive fitness of myeloma upon anti-GPRC5D immunotherapy

**DOI:** 10.1038/s41375-026-02920-7

**Published:** 2026-03-31

**Authors:** Umair Munawar, Johanna Thurner, Silvia Nerreter, Thomas Nerreter, Alexander M. Leipold, Seungbin Han, Christina Verbruggen, Elena Gerhard-Hartmann, Cornelia Vogt, Björn Grams, Shilpa Kurian, Emma Besant, Sabine Roth, Julia Weingart, Patrick Eiring, Marietta Truger, Nazia Afrin, Torsten Steinbrunn, Yoko Tamamushi, Xiang Zhou, Nina Rein, Johanna Lehmann, Max Köppel, Andreas Rosenwald, Claudia Haferlach, Michael Hudecek, Antoine-Emmanuel Saliba, Leo Rasche, Markus Sauer, Hermann Einsele, Bernhard Kuster, Johannes Waldschmidt, K. Martin Kortüm

**Affiliations:** 1 Department of Internal Medicine II, University Hospital of Wuerzburg, Wuerzburg, Germany; 2 School of Life Sciences, Technical University of Munich, Freising, Germany; 3 Helmholtz Institute for RNA-based Infection Research, Helmholtz Centre for Infection Research, Wuerzburg, Germany; 4 Institute of Molecular Infection Biology, University of Wuerzburg, Wuerzburg, Germany; 5 Institute of Pathology, University of Wuerzburg, Wuerzburg, Germany; 6 Department of Biotechnology and Biophysics, University of Wuerzburg, Wuerzburg, Germany; 7 MLL Munich Leukemia Laboratory, Munich, Germany; 8 Mildred Scheel Early Career Center (MSNZ), University Hospital Wuerzburg, Wuerzburg, Germany; 9 Department of Medical Oncology, Dana-Farber Cancer Institute, Harvard Medical School, Boston, USA; 10 INTERACT Advanced Clinician Scientist-Program, University of Wuerzburg, Wuerzburg, Germany

**Keywords:** Myeloma, Translational research

## Abstract

Immunotherapies targeting surface antigens have transformed the treatment landscape of multiple myeloma (MM), with GPRC5D emerging as a promising therapeutic target. Monoallelic loss of *GPRC5D* is frequently observed in newly diagnosed MM patients, and the incidence of acquired *GPRC5D* alterations increases following exposure to GPRC5D-directed therapies. However, the functional consequences of both baseline monoallelic and therapy-induced biallelic *GPRC5D* alterations remain poorly understood. In this study, we modeled monoallelic versus biallelic loss of *GPRC5D* to investigate their impact on MM cell biology and responsiveness to GPRC5D-targeted immunotherapies. Our results demonstrate that monoallelic *GPRC5D* loss in OPM-2 cells reduces surface expression of the antigen and confers resistance to GPRC5D-directed therapies. Complete loss of *GPRC5D* alters the transcriptional state of MM cells and promotes reprogramming of the phosphoproteomic circuitry ultimately resulting in a pro-proliferative chemokine environment. As a result, GPRC5D deficiency increases the basal proliferation rate of MM cells thereby providing a competitive advantage which may further be amplified by selecting these aggressive phenotypes during ongoing treatment with anti-GPRC5D immunotherapies.

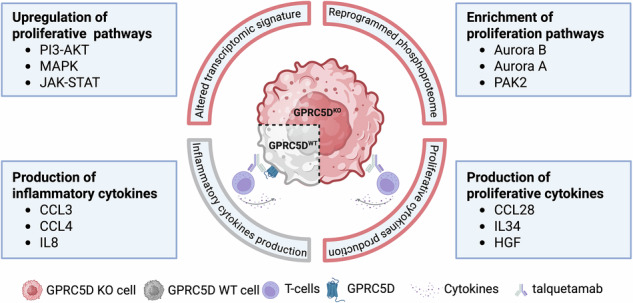

## Introduction

The therapeutic landscape of multiple myeloma (MM) has undergone a paradigm shift with T cell redirecting strategies including bispecific T cell engagers (TCEs) and chimeric antigen receptor T cells (CAR-T) established as integral parts of MM care. Among the emerging targets, G protein-coupled receptor class C group 5 member D (GPRC5D) has gained significant attention as a promising surface antigen in MM [1, 2]. GPRC5D is a seven-transmembrane orphan receptor first identified over two decades ago [3], which exhibits a highly restricted expression profile largely confined to plasma cells and keratinized tissues such as hair follicles and nail beds, with minimal expression in vital organs [4]. This exclusive expression minimizes the risk of widespread off-tumor toxicity and makes it an attractive target for immunotherapeutic intervention. Interestingly, while its clinical value as a therapeutic target is now established, the endogenous ligand of GPRC5D and its biological function in plasma cell biology and MM pathophysiology remains to be clarified.

The clinical efficacy of GPRC5D-targeted immunotherapy is being exemplified by talquetamab, a bispecific antibody that links CD3 on T cells with GPRC5D on MM cells. In the phase 1/2 MonumenTAL-1 study, talquetamab demonstrated highly potent single-agent activity, achieving response rates of approximately 70% in heavily pretreated, triple-class refractory MM patients [5]. Importantly, clinical responses were also observed in patients refractory to B-cell maturation antigen (BCMA)-directed therapies, positioning GPRC5D as a critical alternative or sequential target following BCMA failure. Talquetamab has since received regulatory approval for the treatment of relapsed/refractory MM (RRMM) and is currently available after 3+ and 4+ lines of therapy in Europe and the US, respectively.

Despite this encouraging efficacy, patients continue to relapse, and in contrast to BCMA, where antigen loss is a relatively infrequent mechanism of resistance [6–8], recent data suggest that GPRC5D loss occurs at greater frequency [9–11]. Additionally, epigenetic silencing of the second allele in monoallelic GPRC5D models may further contribute to reduced GPRC5D expression, as previously reported by us and others [12–14]. Whole-genome sequencing analysis prior to talquetamab exposure has revealed that up to 15% of MM patients harbor monoallelic *GPRC5D* deletions [15]. While this represents the highest frequency among all evaluated MM surface antigens, it may be speculated that such monoallelic alterations may serve as a genetic “first hit” that predisposes tumor cells to complete antigen loss under therapeutic pressure. In line with these findings, in the MCARH109 phase I trial of GPRC5D-targeted CAR-T therapy, all relapsed patients (*n* = 6) demonstrated markedly reduced or absent GPRC5D expression [9], underscoring antigen-negative escape as a dominant mechanism of therapeutic resistance.

In the present study, we modeled both monoallelic and biallelic alterations of *GPRC5D* in MM cell lines to explore how mono- vs. biallelic loss of *GPRC5D* affects the efficacy of GPRC5D-targeted immunotherapies. To further investigate the physiological role of GPRC5D, we comprehensively characterized GPRC5D knockout models in the context of T cell redirecting therapies. Our observations provide insight on novel mechanisms of resistance, which will inform future strategies to enhance the durability and safety of GPRC5D-directed treatments in MM.

## Methods

### Cell culture

The MM cell line OPM-2 was purchased from the German Collection of Microorganisms and Cell Cultures (DSMZ). Cells were expanded and frozen as stock and working batch. Cell culture was initiated from working batch and kept in culture using RPMI1640 medium supplemented with 10% FBS, 1 mM Sodium Pyruvate, 2 mM Glutamate, 100U/ml Penicillin, and 100 µg/ml Streptomycin at 37 °C in 5% ambient CO_2_. Cell cultures were renewed every 3 months and regularly tested for mycoplasma contamination.

### CRISPR-Cas9 guide RNA design, generation and screening of KO clones

Guide RNAs targeting *GPRC5D* were generated from the Broad Institute GPP web portal. Oligonucleotides were synthesized (Sigma Aldrich, Table S1), annealed as dsDNA fragments and cloned into a GeneArt CRISPR nuclease vector with OFP Reporter Kit (Thermo Fisher Scientific, A21174) using the manufacturer’s instructions. The gRNA expression plasmids were delivered into the cells using the Neon electroporation system (Thermo Fisher Scientific). Positively transfected cells were sorted 48 h post-electroporation using OFP as a sorting marker via FACS Aria III (Becton-Dickinson). Single-cell derived monocultures were screened for expression using TaqMan (Thermo Fisher Scientific) predesigned probes (Table S2).

### *d*STORM

A PBS-based imaging buffer containing 10 mM cysteamine hydrochloride (M6500-25G, Sigma) at pH 7.4 was used for reversible photoswitching of Alexa Fluor 647 (AF647). *d*STORM measurements were performed via an Olympus IX-71 inverted microscope (Olympus) equipped with an oil immersion objective (PlanApo N 60×1.45 TIRF, Olympus) and a nosepiece stage (IX2-NPS, Olympus)[16, 17]. Detailed methods of imaging and image reconstruction are described in Supplementary Methods.

### Bioluminescence based cytotoxicity assay

Sleeping beauty expression vector (SB) coding for firefly luciferase gene and pTX100 transposase expression plasmid were delivered into the cells via a Neon transfection system. Puromycin (1 µg/ml, 10–14 days) was used as selection marker for stable integration of transgene. For details on cytotoxicity assay setups, see Supplementary Methods.

### Phosphoproteome analysis

Detailed sample preparation, LC-MS3 and protein and peptide quantification are described in Supplementary Methods. Data analysis was performed using the Perseus software [18] (version 1.6.1.1) and Python on identified and quantified protein groups as provided by the “proteinGroups.txt” MaxQuant and the “Phospho(STY)Sites.txt” output files. This data was validated for potential contaminants and reverse hits, and log2 transformation as well as normalization via median centering were performed. Only class I phosphorylation sites with a localization probability of at least 75% were considered. Entries were filtered for at least three valid values per condition, and remaining missing values were not considered for downstream analysis. Two-sample t-tests were performed for 2-group comparisons (permutation-based FDR: 1%, number of randomizations: 250). For enrichment analyses, proteins and pSTY sites were functionally annotated in Perseus, and Fisher exact test was performed using Benjamini-Hochberg correction and an FDR threshold of 0.01.

### scRNA-seq analysis

Sample preparation for scRNA-seq analysis is described in detail in Supplementary Methods. Raw sequencing data were demultiplexed and quantified using CellRanger software suite (v8.0.1, 10x Genomics) with the human reference genome GRCh38 (Ensembl98) for alignment. R (v4.2.3) and Seurat (v5.0.1) were used for downstream analyses. Detailed data analysis is described in Supplementary Methods.

### Clonal competition assays

Cell line models were engineered for stable expression of fluorescent proteins (GFP or RFP) using the SB plasmid system. Clonal competition assays were set up by mixing GPRC5D WT and deficient models at different starting ratios and flow cytometric analysis was performed at regular intervals as described in Supplementary Methods.

### Manufacturing of CAR-T cells

For the generation of human CAR-T cells, healthy donor peripheral blood mononuclear cells (PBMCs) were obtained from leukocyte reduction chambers provided by the Department of Transfusion Medicine of the University Hospital Wuerzburg. A detailed method for CAR-T cell manufacturing is described in Supplementary Methods. CAR-T cells generated from healthy donors were used in allogeneic co-cultures with myeloma cell line targets.

### Cytokine quantification assay

Cells were incubated with various concentrations of talquetamab along with PBMCs or Pan-T cells from healthy donors at an effector:target ratio (E:T ratio) of 4:1 for 20 h. All co-culture assays were performed in an allogeneic setting using healthy donor PBMCs/Pan-T cells as effectors and the OPM-2 myeloma cell line models as targets. Supernatants were collected and cytokine quantification was performed using the ELISA MAX deluxe sets Human IFN-γ (430116) and Human IL-2 (431816) (BioLegend) according to the manufacturer´s instructions. Sciomics performed the comprehensive protein analysis as described in Supplementary Methods.

### Statistics and reproducibility

All in vitro data presented in this study are representative of at least three independent replicates. Healthy T cells for cytotoxicity experiments with TCEs were isolated from two or more different healthy donors. Comparisons between two groups were performed using unpaired *t* test and comparisons of more than two groups were conducted by two-way ANOVA test. All results represent mean values ± standard deviation.

## Results

### Reduced GPRC5D expression in monoallelic *GPRC5D* models is associated with decreased treatment response to talquetamab

**Fig. 1:**
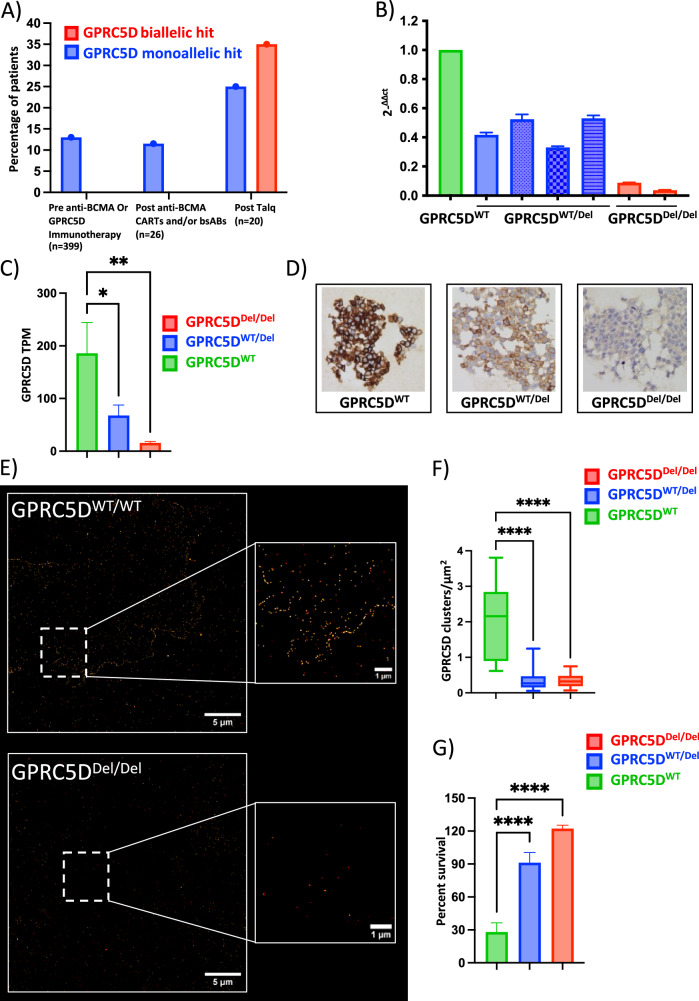
Spectrum of clinical *GPRC5D* alterations and functional characterization of GPRC5D-deficient in vitro models. **A** Frequency and mutational make-up of *GPRC5D* alterations in a MM patient cohort without prior anti-GPRC5D/BCMA exposure (*n* = 399), after anti-BCMA therapy (*n* = 26) and at the time of relapse to anti-GPRC5D bispecific antibody treatment (*n* = 20). Data collected at University of Wuerzburg Medical Center and based on available WGS data. **B** qPCR analysis of OPM-2 MM cell models with monoallelic *GPRC5D* deletion (*GPRC5D*^WT/Del^, blue) and biallelic *GPRC5D* loss (*GPRC5D*^Del/Del^, red) as normalized to *GPRC5D*^WT^ models (green). **C** Transcriptomic analysis represented as transcripts per million (TPM) in *GPRC5D*^WT^ and deficient cell models. **D** Representative images from immunohistochemistry analysis. **E** Representative images of GPRC5D cell models as objectified by *d*STORM microscopy and **F** quantified as clusters/µm^2^. **G** Bioluminescence-based cell survival analysis performed on GPRC5D models treated with 2.5 µg/ml talquetamab for 48 h. *GPRC5D*^WT^ cells are represented in green, *GPRC5D*^WT/Del^ cells are represented in blue and *GPRC5D*^Del/Del^ cells are represented in red. *p < 0.05, **p < 0.005 ***p < 0.001, ****p < 0.0001.

We analyzed an extended MM patient cohort (*n* = 399) prior to treatment with GPRC5D- or BCMA-directed immunotherapies and observed a baseline frequency of 13.0% monoallelic *GPRC5D* alterations. Upon acquired resistance to talquetamab, this frequency increased significantly to 25.0% patients showing monoallelic and an additional 35.0% patients showing biallelic *GPRC5D* loss. However, this frequency remained 11.5% in patients treated with anti-BCMA agents (Fig. 1A). To study the mechanistic impact of *GPRC5D* loss in MM cells, monoallelic and biallelic *GPRC5D* models were generated using OPM-2 and CRISPR-Cas9 technology. RT-PCR profiling showed a 2.1-fold reduction in *GPRC5D* expression in GPRC5D^WT/Del^ cells and a 98% reduction in GPRC5D^Del/Del^ cells (Fig. 1B). Bulk RNA-seq confirmed reduction in *GPRC5D* expression at transcript level with 186 vs. 67.7 (*p* = 0.011) vs. 15.6 (*p* = 0.002) transcripts per million (TPM) in GPRC5D^WT^ vs. GPRC5D^WT/Del^ vs. GPRC5D^Del/Del^ models (Fig. 1C) and was confirmed by digital droplet PCR (ddPCR) (Figure S1) and Sanger sequencing. IHC and *d*STORM ultra high-resolution single molecule microscopy were subsequently employed to confirm the reduction in GPRC5D expression at protein level (Fig. 1D). *d*STORM revealed a significant reduction in GPRC5D surface expression on GPRC5D^WT/Del^ (0.35 ± 0.02 clusters/µm^2^) vs. WT cells (1.89 ± 0.10 clusters/µm^2^, *p* < 0.0001). GPRC5D^Del/Del^ models had barely detectable GPRC5D expression with 0.03 ± 0.07 clusters/µm^2^ (*p* < 0.0001, Fig. 1E, F). To investigate if sensitivity to GPRC5D-directed treatments would decline with reduced GPRC5D expression, cells were next treated with 2.5 µg/ml talquetamab at an E:T ratio of 10:1 for 48 h. GPRC5D^WT/Del^ cells showed a 3.3-fold increase in talquetamab resistance as compared to GPRC5D^WT^ cells (*p* < 0.0001) whereas complete resistance was observed for GPRC5D^Del/Del^ models (*p* < 0.0001) (Fig. 1G). Different E:T ratios were tested, with no significant variation in outcome observed across tested conditions (Fig. S2).

These data indicate that even monoallelic loss of *GPRC5D* leads to therapeutically relevant reductions in GPRC5D expression and drives partial resistance to talquetamab in preclinical cell line models.

### GPRC5D deficiency increases baseline proliferation of MM cells

**Fig. 2:**
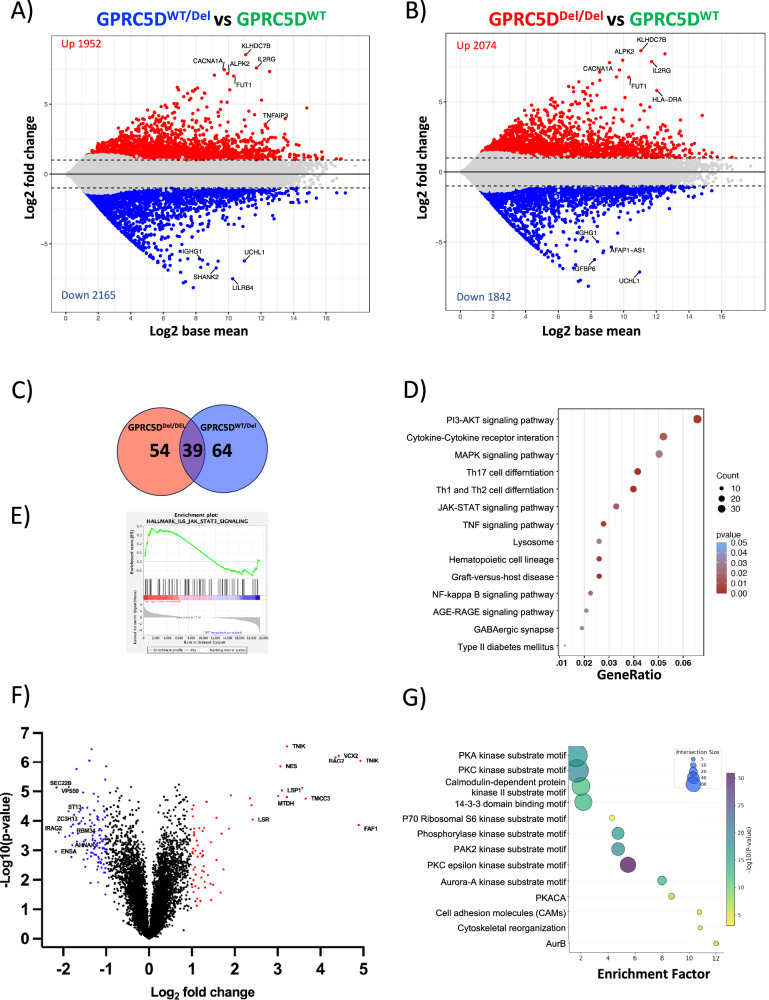
Transcriptomic and phosphoproteomic landscape of *GPRC5D*^WT^ and deficient models. Plots indicating gene expression differences between GPRC5D^WT/Del^ vs. GPRC5D^WT^ models (**A**) and between GPRC5D^Del/Del^ vs. GPRC5D^WT^ models (**B**). Differentially expressed genes with an adjusted *p* value < 0.05 and a log 2FC > │1│ are depicted in colors. **C** Venn diagram of overlapping DEG in GPRC5D^Del/Del^ and GPRC5D^WT/Del^ cell models^.^ **D** Dot plot of enriched pathways overlapping between GPRC5D^Del/Del^ and GPRC5D^WT/Del^ models. **E** Enrichment plots of JAK-STAT3 pathway from GSEA. Enrichment profile is indicated as green line. **F** Volcano plot showing differentially expressed phosphosites between GPRC5D^Del/Del^ and GPRC5D^WT^ models at a log 2FC > │2│changed. **G** Perseus analysis depicting enriched pathways at phosphoproteomic level between GPRC5D^Del/Del^ and GPRC5D^WT^ models.

**Fig. 3:**
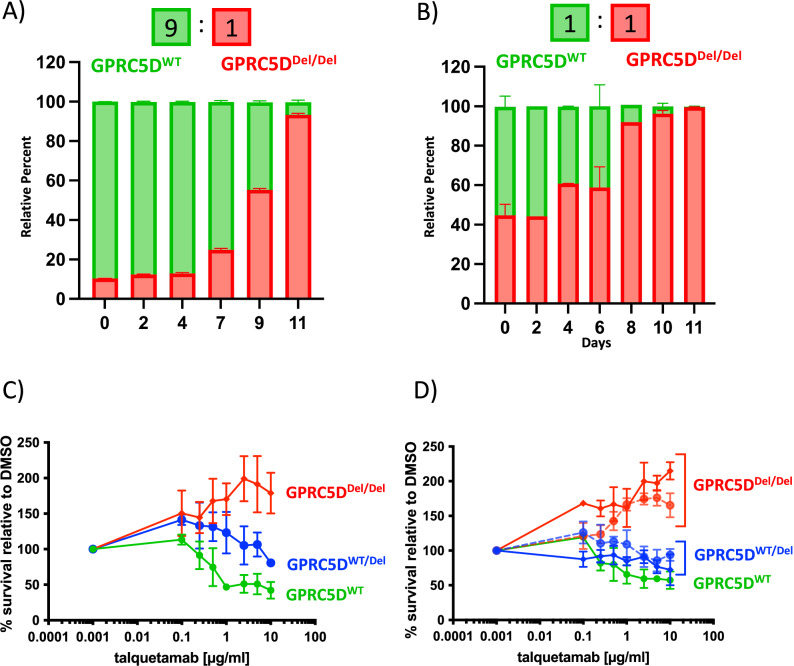
Functional impact of *GPRC5D* alterations. Clonal competition assay showing the selection dynamics of co-cultures over 11 days with a 9:1 (**A**) and 1:1 (**B**) starting ratio for GPRC5D^WT^ vs. GPRC5D^Del/Del^ cells. **C** Talquetamab titration curve performed on GPRC5D models. Cells were treated with talquetamab for 48 h and luciferin-based cytotoxicity assay was performed. T cells from three different healthy donors were used as effector cells. **D** Additionally, two clones for each type of genetic alteration were treated with talquetamab. *GPRC5D*^WT^ cells are represented in green. *GPRC5D*^WT/Del^ cells are represented in blue and *GPRC5D*^Del/Del^ cells are represented in red.

To investigate the biological implications of *GPRC5D* loss on the cellular homeostasis of MM cells, we performed bulk RNA-seq on GPRC5D^WT^, GPRC5D^WT/Del^ and GPRC5D^Del/Del^ cell line models. Differential expression analysis revealed 1952 upregulated and 2165 downregulated genes in GPRC5D^WT/Del^, and 2074 upregulated and 1842 downregulated genes in GPRC5D^Del/Del^ as compared to GPRC5D^WT^ cells (*padj* = 0.05) (Fig. 2A, B). Downstream characterization by gene set enrichment analysis (GSEA) confirmed a total of 39 pathways to be consistently dysregulated in both single- and double-hit GPRC5D models (Fig. 2C), whereof, most prominently, we observed enrichment of pro-proliferative signaling programs such as PI3K, MAPK, and JAK-STAT pathways in GPRC5D-deficient vs. -WT models (Fig. 2D, E). Changes at protein level were assessed by mass spectrometry (MS)- based proteomics, with quantification of phosphoproteome levels using TMT as a labeling reagent. This unbiased approach corroborated our findings by recapitulating a pattern of increased proliferation and cell cycling in GPRC5D^Del/Del^ vs. GPRC5D^WT^ cells, including functional enrichment of potentially targetable kinase motifs such as Aurora B (EF = 12.03, *FDR* < 0.0001), Aurora A (EF = 7.99, *FDR* < 0.0001) and PAK2 substrates (EF = 4.74, *FDR* < 0.0001) (Fig. 2F, G), and additional enrichment of other kinases such as p70 ribosomal S6 kinase (EF = 4.2, *FDR* < 0.0001), calmodulin dependent protein kinase II (EF = 1.9, *FDR* = 0.01) PKACA (EF 8.9, *FDR* < 0.0001) and PKC epsilon kinase (EF = 5.4, *FDR* < 0.0001), the latter all being involved in the cellular proliferation signaling (Fig. 2G). To determine if the observed transcriptomic and phosphoproteomic alterations have an impact on the functional properties of MM cells, we next performed clonal competition assays between GPRC5D^WT^ and GPRC5D^Del/Del^ cells. In a co-culture starting with a 1:9 Del/Del:WT ratio, GPRC5D^Del/Del^ cells showed a clear growth advantage, reaching over 90% of the entire population by day 11 (Fig. 3A). The dominance of GPRC5D^Del/Del^ was even more pronounced when starting at a 1:1 ratio, exceeding 90% of the whole population by day 8 (Fig. 3B).

Collectively, these findings demonstrate that GPRC5D loss perturbs MM cell homeostasis through transcriptomic and phosphoproteomic networks that favor increased proliferative capacity as compared to GPRC5D^WT^ cells.

### Talquetamab selects for highly proliferative phenotypes in absence of GPRC5D expression

To further investigate the effects of talquetamab treatment on GPRC5D-deficient cells, we treated our models with talquetamab. Unexpectedly, and while GPRC5D^WT^ cells exhibited a clear dose-dependent killing upon talquetamab exposure, the proliferative capacity in GPRC5D^Del/Del^ models was even further enhanced by the presence vs. absence of talquetamab treatment (Fig. 3C). This gain in proliferation was not observed when cells were incubated with talquetamab in absence of T cells (Fig. S3). To rule out a clone-specific artifact, we performed talquetamab titrations across multiple *GPRC5D* clones harboring either monoallelic or biallelic alterations and observed a similar proliferative response across all models (Fig. 3D). Notably, the activity of classical anti-MM therapies such as melphalan, alkylating agents, bortezomib, or lenalidomide remained unaltered (Fig. S4).

Potential off-target effects of talquetamab were ruled out by investigating alternative binding of talquetamab on GPRC5D-deficient MM cells by studying fluorochrome-labeled talquetamab in *d*STORM microscopy (Fig. 1E, F). To investigate putative mechanisms, by which talquetamab treatment could potentially result in increased proliferation of GPRC5D^Del/Del^ MM cells, we next performed mass spectrometry analysis in talquetamab-treated GPRC5D^Del/Del^ versus GPRC5D^WT^ models. This comparison revealed 83 up- and 99 downregulated phosphosites in talquetamab-treated GPRC5D^Del/Del^ models (Fig. S5). These mostly overlapped with those seen in talquetamab-naïve GPRC5D^Del/Del^ models (Fig. 2F).

Manual enrichment examination of the phosphosites did not result in clear evidence for an alteration of the phosphoproteome through talquetamab treatment. This suggests that the proliferative advantage conferred by talquetamab may occur independently of broad phosphoproteomic changes or that such changes were below the limit of detection, indicating that the additional talquetamab-induced stimulus on the proliferation of GPRC5D^Del/Del^ cells is not intrinsically linked to potential off-target effects of talquetamab, but seems to rather arise from the competitive advantage of GPRC5D^Del/Del^ cells after elimination of GPRC5D^WT^ cells, with more favorable growth conditions, including cytokine and nutrient supply for the remaining GPRC5D^Del/Del^ clones.

### Talquetamab alters T cell transcriptome in presence of GPRC5D on MM cells

**Fig. 4:**
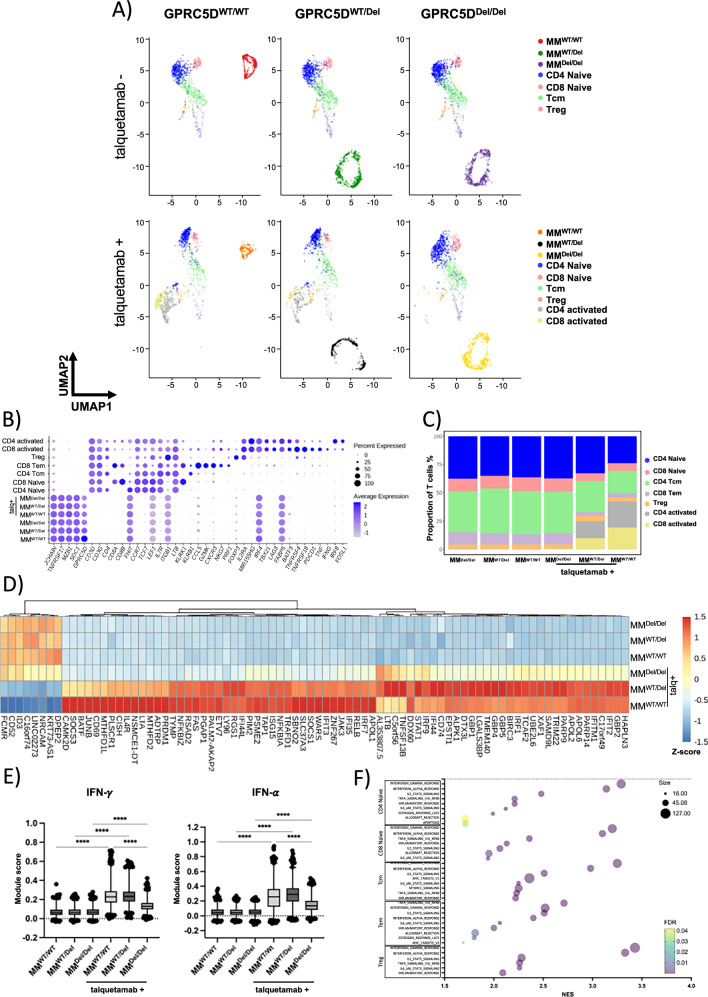
Single-cell RNA-seq of cocultures to identify transcriptional T cell responses to talquetamab and GPRC5D. **A** UMAP representation of single-cell transcriptomes of T cell/MM coculture cells stratified by MM cell-line and talquetamab treatment with cells colored according to their annotation. **B** Dotplot of scaled expression (color) of selected marker genes used for annotation of MM cells and T cell subtypes. Dot size depicts the percentage of non-zero expressing cells. **C** Relative abundance of T cell subpopulations across coculture conditions. **D** Heatmap showing z-scores of mean log-normalized expression for differentially expressed genes (padj <0.001, absolute avg_log2FC > 2) in T cells between talquetamab and control across all coculture conditions. **E** Boxplots depicting single-cell IFN-γ (left) and IFN-α (right) module scores across coculture conditions. The center line indicates the median, the box limits indicate the upper and lower quantiles, and the whiskers indicate 1.5× interquartile range. Statistical significance was determined using two-way ANOVA (**** *p* value < 0.001). **F** GSEA results for upregulated genes within shared T cell subpopulations between talquetamab and control in GPRC5D^WT^.

To better understand the impact of talquetamab on T cells in the presence of GPRC5D on MM cells, we performed CITE-seq. T cells were cocultured with or without talquetamab. We confirmed our previous observation that loss of even a single GPRC5D allele results in transcriptomic alterations in MM cells as seen by entirely different clustering profiles of GPRC5D^WT^ and deficient cells (Fig. 4A).

Based on the expression of canonical marker genes, we identified CD4 and CD8 naïve T cells, CD4 central memory T cells (CD4 Tcm), CD8 effector memory T cells (CD8 Tem) and CD4 regulatory T cells (Treg). In addition, we identified CD4 and CD8 T cells marked by expression of activation genes (*IL2RA, TNFRSF4, TNFRSF18*), as well as *TNF* and *IFNG* (Fig. 4B). As expected, activated T cells were only observed when talquetamab was added to the coculture with GPRC5D^WT^ or GPRC5D^WT/Del^ MM cells, but not with GPRC5D deficient MM cells (Fig. 4A, C).

We assessed gene expression changes in the T cell populations induced by talquetamab, cocultured with GPRC5D expressing MM cells (Table S3). A significant upregulation of interferon stimulated genes (*GBP2, GBP4, GBP5, IFI44, IFIT2, ISG15*) was found in T cells cocultured with GPRC5D expressing cells and talquetamab, while interferon signaling genes (*STAT1, IRF1*) were already induced by talquetamab alone (Fig. 4D). Scoring of interferon responses across T cells confirmed an increase in T cells cocultured with talquetamab that was increased in presence of GPRC5D expressing MM cells (Fig. 4E). We also identified genes which were only upregulated in presence of GPRC5D and talquetamab, including *SOCS3, CD69, JUNB, BATF, CISH*, and *PRDM1*, indicating a unique activation status. Notably, these responses were markedly pronounced in coculture with GPRC5D^WT^ as compared to GPRC5D^WT/Del^ MM cells (Fig. 4D).

Gene set enrichment analysis (GSEA) confirmed upregulation of interferon responses (IFN-γ: NES 2.97, FDR 0.0; IFN-α: NES 2.87, FDR 0.0), and further showed upregulation of IL2-STAT5 signaling (NES 2.13, FDR 0.0), TNFA signaling (NES 2.23, FDR 0.0) and inflammatory response (NES 2.08, FDR 0.0) in T cells after coculture with GPRC5D expressing MM cells and talquetamab as compared to cocultures without talquetamab (Fig. 4F). Of note, T cells did not respond to presence or absence of GPRC5D on MM cells in the absence of talquetamab, and their transcriptomic profile remained unchanged (Table S4).

Our findings demonstrate that talquetamab upregulates the expression of interferon signaling genes in T cells and while this response is enhanced when GPRC5D is present on MM cells, it also occurs in KO models. Moreover, talquetamab alters the activation profile across T cells subsets and both abundance and distribution of different T cells subpopulations is changed by GPRC5D status of MM cells in coculture.

### Talquetamab treatment alters chemokine profile in T cell co-culture with GPRC5D-deficient myeloma cells

**Fig. 5:**
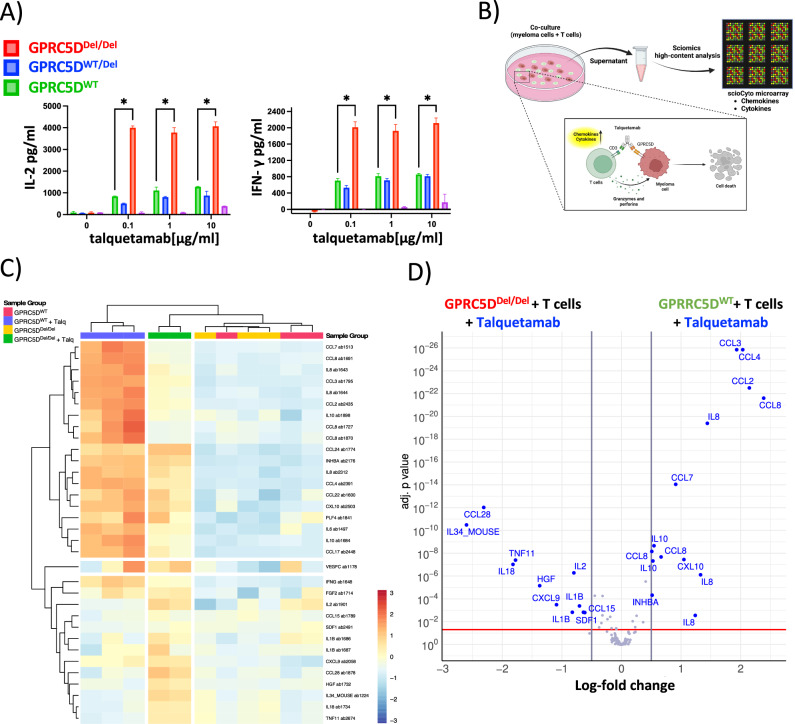
Cytokine quantification for functional profiling of T cells in the presence vs. absence of GPRC5D. **A** Bar graphs representing the levels of IL-2 and IFN-γ in the supernatant. GPRC5D cell models (WT and knock-out) were treated with different talquetamab concentrations for 24 h and the supernatants were analyzed for cytokine production. T cells from two different healthy donors were used as effector cells (negative control is represented in magenta). **B** Graphical representation of experimental setup for high-content chemokine profiling. **C** Heatmap of 120 chemokines analyzed by scioCyto microarray assay. **D** Volcano plot showing differentially produced cytokines in GPRC5D^Del/Del^ and GPRC5D^WT^ models. Differentially produced cytokines with an adjusted *p* < 0.05 and a log 2FC > │0.5│ are depicted in blue color.

To account for talquetamab-induced alterations within the chemokine niche, we next characterized the supernatants of T cell/GPRC5D^Del/Del^ co-cultures in the presence vs. absence of talquetamab. After 20 h of incubation, we observed a significantly elevated secretion of IL-2 and IFN-γ in co-cultures with vs. without talquetamab (Fig. 5A). An expanded screen covering 120 cytokines by high-content chemokine profiling (Fig. 5B) revealed 21 differentially expressed cytokines in GPRC5D^WT^ and 28 in GPRC5D^Del/Del^ models upon exposure to talquetamab treatment, with all relative expression levels of cytokines being summarized in Fig. 5C. Specifically, pro-inflammatory cytokines such as CCL3, CCL4, CCL8, and IL-8 were enriched in talquetamab-treated GPRC5D^WT^ co-cultures, whereas cytokines associated with cell growth and proliferation, including CCL28, IL-34, HGF, and CXCL9, were overexpressed in talquetamab-exposed GPRC5D-deficient co-cultures (Fig. 5D).

These findings indicate that T cells produce distinct cytokine profiles depending on the GPRC5D expression status of MM cells and the respective co-treatment with talquetamab. This differential cytokine milieu may contribute to the proliferative phenotype observed in GPRC5D-deficient models, highlighting a potential interplay between immune activation and tumor cell-intrinsic responses in the context of GPRC5D deficiency.

### GPRC5D-deficient MM cells show comparable competitive advantage with CAR-T and MOCK T cells

**Fig. 6:**
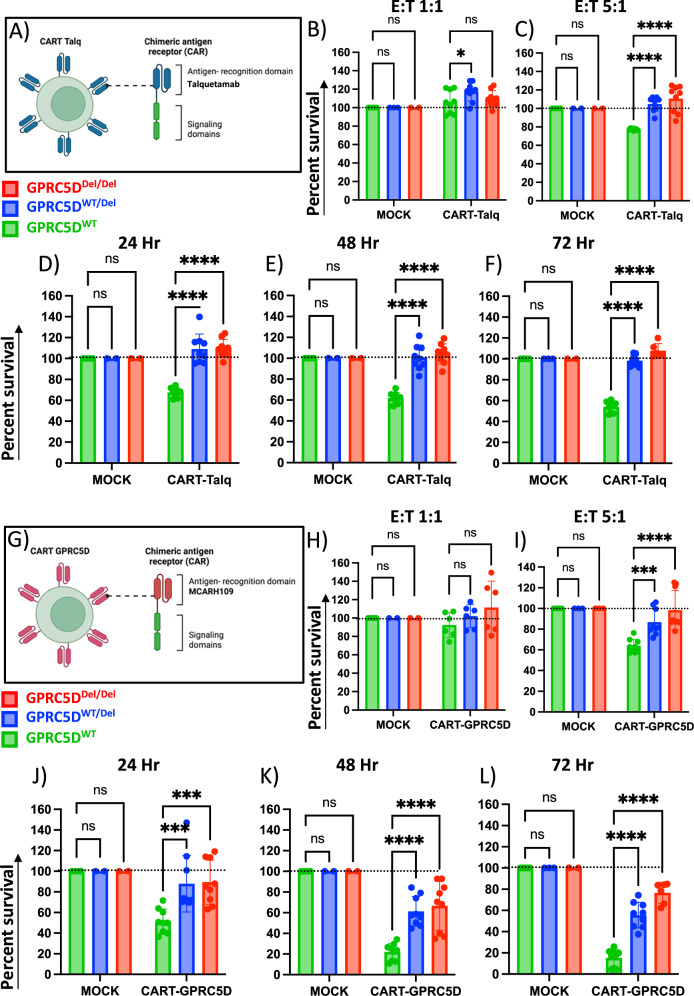
Sensitivity of GPRC5D models towards anti-GPRC5D CAR-T cells. **A** Graphical design of CAR-T-Talq cells. Killing efficacy of CAR-T-Talq CAR-T cells after co-incubation with GPRC5D cell models at E:T ratios of 1:1 (**B**), 5:1 (**C**) and 10:1 (**D**) after 24 h of incubation. CAR-T-Talq killing efficacy at 48 h (**E**) and 72 h (**F**) using E:T ratios of 10:1. Data normalized to MOCK T cells. **G** Graphical design of CAR-T-GPRC5D cells. Killing efficacy of CAR-T-GPRC5D CAR-T cells after co-culture with GPRC5D cell models at E:T ratio of 1:1 (**H**), 5:1 (**I**) and 10:1 (**J**) after 24 h of incubation. CAR-T-Talq killing efficacy at 48 h (**K**) and 72 h (**L**) using E:T ratios of 10:1. *GPRC5D*^WT^ cells are represented in green. *GPRC5D*^WT/Del^ cells are represented in blue and *GPRC5D*^Del/Del^ cells are represented in red. Data normalized to MOCK T cells. *p < 0.05, **p < 0.005 ***p < 0.001, ****p < 0.0001.

**Fig. 7:**
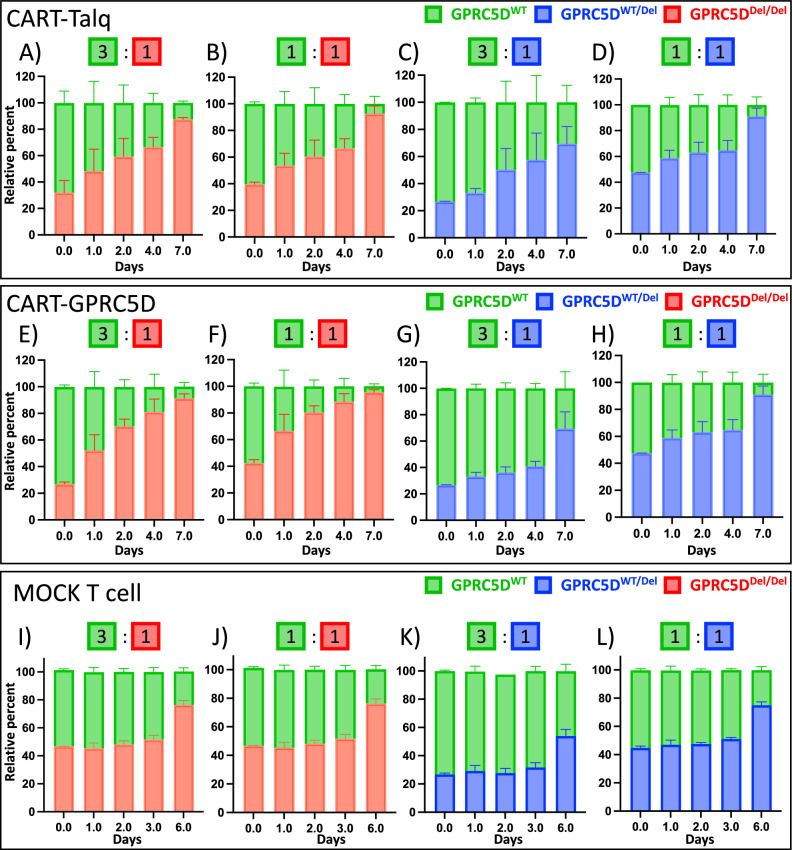
Clonal competition assays performed under selection pressure with anti-GPRC5D CAR-T cells. Clonal competition assays were performed with CAR-T-Talq (**A–D**), CAR-T-GPRC5D (**E–H**), and MOCK T cells (**I–L**) at E:T ratios of 1:1 with either *GPRC5D*^Del/Del^ or *GPRC5D*^WT/Del^ mixed with *GPRC5D*^WT^ at starting ratios of 1:3 or 1:1.

In a next step, we tested the proliferative advantage of GPRC5D^Del/Del^ models in the context of anti-GPRC5D CAR-T cells. First, an in-house CAR-T product which harbors an antigen recognition domain identical to talquetamab (CAR-T-Talq, Fig. 6A), induced dose-dependent killing of GPRC5D^WT^ at E:T ratios of 5:1 and 10:1 and after incubation periods of 24, 48, and 72 h, whereas the same product expectedly had no cytolytic effect in GPRC5D^Del/Del^ and GPRC5D^WT/Del^ models (Fig. 6B–F). To validate these findings, a second CAR construct with identical antigen recognition as in MCARH109 (CAR-T-GPRC5D) was tested and induced similar dose-dependent cell killing of GPRC5D^WT^ cells (Fig. 6G–J). GPRC5D^WT/Del^ and GPRC5D^Del/Del^ models displayed significantly reduced sensitivity across all E:T ratios. Although prolonged co-culture (48–72 h) improved the cytolytic activity against WT cells, GPRC5D-deficient models consistently maintained an overall insensitive profile (Fig. 6K, L). A significant reduction in IFN-*γ* production by CAR-T cells was observed after coculture with GPRC5D deficient MM cell models compared to WT cells (Fig. S6). Clonal competition assays were next conducted with GPRC5D^WT^ and GPRC5D^Del/Del^ cells at different ratios (3:1 and 1:1). Upon selective pressure with CAR-T-Talq (Fig. 7A–D) or CAR-T-GPRC5D (Fig. 7E–H), both CAR-Ts preferentially eliminated WT cells, resulting in a relative enrichment of GPRC5D-deficient clones over time. Interestingly, simultaneous co-culture experiments using MOCK T cells, that had been cultured analogously to both CAR-T products, led to a delayed but significant increase of GPRC5D^Del/Del^ cells within the competition assay by day 6 of co-culture (Fig. 7I–L).

Taken together, this data suggests that target antigen loss depicts an expected immune evasion mechanism which can equally occur in MM cells with bi- but also monoallelic *GPRC5D* alterations. Beyond antigen loss, GPRC5D deficiency however also increased the basal proliferation rate of MM cells thereby providing a competitive advantage which was further amplified by selecting these aggressive phenotypes during ongoing treatment with anti-GPRC5D immunotherapies.

## Discussion

In this study, we provide mechanistic insight into the biological implications of *GPRC5D* loss on cellular homeostasis and target evasion of MM tumor cells. First, we report on the relevance of monoallelic *GPRC5D* alterations on GPRC5D surface expression of MM cells which subsequently lead to partial resistance to GPRC5D-directed immunotherapeutic intervention. Building on our previous report [15], we observed a baseline frequency of 13.0% monoallelic *GPRC5D* alterations in an expanded cohort of 399 MM patients prior to treatment with GPRC5D- or BCMA-targeted immunotherapies. Following therapeutic exposure, this frequency remained 11.5% in patients treated with anti-BCMA agents but increased significantly in those with acquired resistance to the anti-GPRC5D bispecific antibody talquetamab, with approximately 25.0% showing monoallelic and an additional 35.0% showing biallelic *GPRC5D* losses. The loss of GPRC5D, even at partial extent, compromised efficacy of GPRC5D-directed CAR-T and bispecific therapies, particularly at low E:T ratios with selective enrichment of these cells over time suggesting immune-driven clonal selection.

To move beyond genomic antigen loss, a resistance mechanism which is now well established in the context of talquetamab and anti-GPRC5D CAR-T cells [9, 10], we next investigated the adaptive changes following loss of GPRC5D and, surprisingly, discovered a consistent increase in the baseline proliferative capacity of GPRC5D-deficient MM cells, which was driven by consistent upregulation of proliferation-associated pathways at both transcriptomic and phosphoproteomic levels. It may be hypothesized that such broad rewiring of the transcriptomic and proteomic circuitry in GPRC5D-deficient cells including upregulation of pathways like IL-6/STAT3 and MAPK, may depict a compensatory mechanism to maintain growth-promoting signaling and thereby compensate for antigen loss. This is consistent with findings by Ma et al. [14] who reported transcriptional and epigenetic remodeling in relapsed GPRC5D-negative cells, including altered chromatin accessibility at the *GPRC5D* locus. We observed that GPRC5D deficient cells show reduced sensitivity to MEK inhibitor Trametinib (Fig. S7), as reported by Novoplansky et al. [19] likely due to the upregulation of PI3K-AKT pathway. These adaptations confer a fitness advantage, particularly under immune pressure, and may drive more aggressive disease phenotypes, similar to a recent study linking *GPRC5D* silencing to more aggressive, therapy-resistant disease phenotypes [12].

CITE-seq performed on T cells revealed that talquetamab leads to the upregulation of interferon responding genes in T cells irrespective of the presence of target cells. Interestingly, our data suggest a cytokine-mediated selection pressure in GPRC5D-deficient models with increased secretion of IL-2, IFN-γ, IL-6, IL-10, and HGF when T cell are engaging with GPRC5D-low target cells, possibly due to inefficient synapse formation or tonic activation with indefinite autocrine and paracrine activation [20–22]. This indicates an important interplay between antigen expression, T cell function, and cytokine signaling, which may be exploited therapeutically, for example, by combining GPRC5D-directed therapy with IL-6 or JAK/STAT inhibitors [23].

A key limitation of this study is the use of a single cell line, OPM-2, which may not fully represent the intrinsic heterogeneity of MM. While this may constrain the generalizability of our findings, OPM-2 was chosen due to its high GPRC5D expression, a feature directly relevant to the biological question under investigation. As the establishment of a new cell line was constrained by technical limitations, we instead employed a reverse strategy involving overexpression of GPRC5D in three distinct cell lines/models (AMO-1, OPM-2 and OPM-2^KO^). We found that overexpression of GPRC5D in AMO-1 cell line imparted proliferative disadvantage compared to AMO-1^WT^ cells in clonal competition assays for up to 10 days followed by a state of equilibrium (Fig. S8 A,B). Similarly, both OPM-2^WT^ and OPM-2^KO^ cells with exogenous GPRC5D overexpression showed attenuated proliferation potential compared to respective controls for up to 7 days (Fig. S8 C,D). This compromised proliferation fitness diminished in long term competition assays due to the enhanced cell death of the cells after 7 days of coculture (Fig. S8 E). In addition, our T-cell–myeloma co-culture experiments were performed in an allogeneic setting using healthy donor effector cells with myeloma cell line targets. While this design enables controlled assessment of antigen-dependent effects across isogenic target genotypes, it may not fully capture patient-specific immune fitness and microenvironmental suppression. Future validation in autologous patient-derived co-culture systems will be important to translate these findings to individual patient contexts.

Our findings have direct clinical implications, including the need for more frequent screening of GPRC5D mutants to detect early development of target evasion. Second, dual-targeting CARs or trispecific antibodies (e.g., targeting BCMA and GPRC5D) may prevent clonal escape by targeting vulnerable subpopulations before they expand [24, 25]. Independent from target evasion, our data further leverages on the putative biological functions of GPRC5D, given that its absence in MM cell line models results in immediate shifts within the transcriptomic and phosphoproteomic network which ultimately results in a proliferative fitness advantage for GPRC5D-deficient over-wildtype cells.

Future studies will have to confirm if this competitive advantage of GPRC5D-deficient cells also holds true in the context of T cell agnostic treatment modalities, including antibody-drug conjugates which are now also becoming available against GPRC5D (AZD0305 and LM-305) [26, 27]. Similarly, activation of compensatory signaling pathways may provide secondary vulnerabilities to counteract the induced proliferative advantages of GPRC5D-deficient cells [20, 21].

In summary, our study delineates the dual impact of GPRC5D loss in MM, linking it to both resistance to GPRC5D-targeted immunotherapy and enhanced tumor cell fitness via proliferative reprogramming. Notably, even monoallelic loss was functionally significant and may serve as an early indicator of GPRC5D treatment resistance.

## Supplementary information

Supplemental material

Supplemental tables

## Data Availability

The mass spectrometry proteomics data and complete MaxQuant search results were uploaded to the ProteomeXchange Consortium (http://www.proteomexchange.org/) via the MassIVE repository with the data set identifier MSV000097698. RNA-seq data is available at following accession number (10.5281/zenodo.18619361). Other data generated in this study are available from corresponding authors upon request.

## References

[1] Rodriguez-Otero P, van de Donk NWCJ, Pillarisetti K, Cornax I, Vishwamitra D, Gray K, et al. GPRC5D as a novel target for the treatment of multiple myeloma: a narrative review. Blood Cancer J. 2024;14:24. 38307865 10.1038/s41408-023-00966-9 PMC10837198

[2] Nath K, Costa BA, Mailankody S. GPRC5D as a novel immunotherapeutic target in multiple myeloma. Nat Rev Clin Oncol. 2023;20:281–2. 36725915 10.1038/s41571-023-00735-4

[3] Bräuner-Osborne H, Jensen AA, Sheppard PO, Brodin B, Krogsgaard-Larsen P, O’Hara P. Cloning and characterization of a human orphan family C G-protein coupled receptor GPRC5D. Biochim Biophys Acta. 2001;1518:237–48. 11311935 10.1016/s0167-4781(01)00197-x

[4] Inoue S, Nambu T, Shimomura T. The RAIG family member, GPRC5D, is associated with hard-keratinized structures. J Invest Dermatol. 2004;122:565–73. 15086536 10.1046/j.0022-202X.2004.12628.x

[5] Rasche L, Schinke C, Touzeau C, Minnema MC, van de Donk NW, Rodríguez-Otero P, et al. Long-term efficacy and safety results from the phase 1/2 MonumenTAL-1 study of talquetamab, a GPRC5D× CD3 bispecific antibody, in patients with relapsed/refractory multiple myeloma (RRMM). Clin Lymphoma Myeloma Leukemia. 2024;24:S561-S562.

[6] Da Vià MC, Dietrich O, Truger M, Arampatzi P, Duell J, Heidemeier A, et al. Homozygous BCMA gene deletion in response to anti-BCMA CAR T cells in a patient with multiple myeloma. Nat Med. 2021;27:616–9. 33619368 10.1038/s41591-021-01245-5

[7] Samur MK, Fulciniti M, Aktas Samur A, Bazarbachi AH, Tai Y-T, Prabhala R, et al. Biallelic loss of BCMA as a resistance mechanism to CAR T cell therapy in a patient with multiple myeloma. Nat Commun. 2021;12:868. 33558511 10.1038/s41467-021-21177-5 PMC7870932

[8] Firestone RS, Socci ND, Shekarkhand T, Zhu M, Qin WG, Hultcrantz M, et al. Antigen escape as a shared mechanism of resistance to BCMA-directed therapies in multiple myeloma. Blood. 2024;144:402–7. 38728378 10.1182/blood.2023023557 PMC11302451

[9] Mi X, Penson A, Abdel-Wahab O, Mailankody S. Genetic Basis of Relapse after GPRC5D-Targeted CAR T Cells. N Engl J Med. 2023;389:1435–7. 37819961 10.1056/NEJMc2308544 PMC11660085

[10] Lee H, Ahn S, Maity R, Leblay N, Ziccheddu B, Truger M, et al. Mechanisms of antigen escape from BCMA-or GPRC5D-targeted immunotherapies in multiple myeloma. Nat Med. 2023;29:2295–306. 37653344 10.1038/s41591-023-02491-5 PMC10504087

[11] Mog BJ, Marcou N, DiNapoli SR, Pearlman AH, Nichakawade TD, Hwang MS, et al. Preclinical studies show that Co-STARs combine the advantages of chimeric antigen and T cell receptors for the treatment of tumors with low antigen densities. Sci Transl Med. 2024;16:eadg7123. 38985855 10.1126/scitranslmed.adg7123 PMC12226805

[12] Derrien J, Gastineau S, Frigout A, Giordano N, Cherkaoui M, Gaborit V, et al. Acquired resistance to a GPRC5D-directed T-cell engager in multiple myeloma is mediated by genetic or epigenetic target inactivation. Nat Cancer. 2023;4:1536–43. 37653140 10.1038/s43018-023-00625-9

[13] Han S, Munawar U, Haertle L, Vogt C, Nerreter S, Teufel E, et al. Functional characterization of GPRC5D alteration and its impact on talquetamab resistance in relapsed/ refractory multiple myeloma. Blood. 2023;142:3323.

[14] Ma S, Xia J, Zhang M, Li W, Xiao M, Sha Y, et al. Genetic and epigenetic mechanisms of GPRC5D loss after anti-GPRC5D CAR T-cell therapy in multiple myeloma. Blood. 2025;146:178–90. 40090012 10.1182/blood.2024026622 PMC12782979

[15] Truger MS, Duell J, Zhou X, Heimeshoff L, Ruckdeschel A, John M, et al. Single-and double-hit events in genes encoding immune targets before and after T cell–engaging antibody therapy in MM. Blood Adv. 2021;5:3794–8. 34471932 10.1182/bloodadvances.2021004418 PMC8679680

[16] Heilemann M, Van De Linde S, Schüttpelz M, Kasper R, Seefeldt B, Mukherjee A, et al. Subdiffraction-resolution fluorescence imaging with conventional fluorescent probes. Angew Chem Int Ed. 2008;47:6172–6. 10.1002/anie.200802376 18646237

[17] Van de Linde S, Löschberger A, Klein T, Heidbreder M, Wolter S, Heilemann M, et al. Direct stochastic optical reconstruction microscopy with standard fluorescent probes. Nat Protoc. 2011;6:991–1009. 21720313 10.1038/nprot.2011.336

[18] Tyanova S, Temu T, Sinitcyn P, Carlson A, Hein MY, Geiger T, et al. The Perseus computational platform for comprehensive analysis of (prote) omics data. Nat methods. 2016;13:731–40. 27348712 10.1038/nmeth.3901

[19] Novoplansky O, Shnerb AB, Marripati D, Jagadeeshan S, Abu Shareb R, Conde-López C, et al. Activation of the EGFR/PI3K/AKT pathway limits the efficacy of trametinib treatment in head and neck cancer. Mol Oncol. 2023;17:2618–36. 37501404 10.1002/1878-0261.13500 PMC10701778

[20] Mohamed AH, Ahmed AT, Al Abdulmonem W, Bokov DO, Shafie A, Al-Hetty H, et al. Interleukin-6 serves as a critical factor in various cancer progression and therapy. Med Oncol. 2024;41:182. 38900329 10.1007/s12032-024-02422-5

[21] Zhang M, Chen J, Zhang H, Dong H, Yue Y, Wang S. Interleukin-10 increases macrophage-mediated chemotherapy resistance via FABP5 signaling in multiple myeloma. Int Immunopharmacol. 2023;124:110859. 37666065 10.1016/j.intimp.2023.110859

[22] Börset M, Hjorth-Hansen H, Seidel C, Sundan A, Waage A. Hepatocyte growth factor and its receptor c-met in multiple myeloma. Blood. 1996;88:3998–4004. 8916966

[23] Shah JJ, Feng L, Thomas SK, Berkova Z, Weber DM, Wang M, et al. Siltuximab (CNTO 328) with lenalidomide, bortezomib and dexamethasone in newly-diagnosed, previously untreated multiple myeloma: an open-label phase I trial. Blood Cancer J. 2016;6:e396. 26871714 10.1038/bcj.2016.4 PMC4771967

[24] Cohen YC, Magen H, Gatt M, Sebag M, Kim K, Min CK, et al. Talquetamab plus Teclistamab in Relapsed or Refractory Multiple Myeloma. N Engl J Med. 2025;392:138–49. 39778168 10.1056/NEJMoa2406536

[25] Zhou D, Sun Q, Xia J, Gu W, Qian J, Zhuang W, et al. Anti-BCMA/GPRC5D bispecific CAR T cells in patients with relapsed or refractory multiple myeloma: a single-arm, single-centre, phase 1 trial. Lancet Haematol. 2024;11:e751–e60. 39059405 10.1016/S2352-3026(24)00176-5

[26] Lim S-L, Augustson B, Mian HS, Stadtmauer EA, Iida S, Slade M, et al. A Phase I/II study of AZD0305, a novel antibody-drug conjugate (ADC) targeting GPRC5D, in patients with relapsed/refractory multiple myeloma (RRMM). Blood. 2024;144 Suppl 1:2000.2-2.

[27] Huang W, Luo J, Li Y, Fei D, Qin X, Li R Abstract 6020: Preclinical activity of LM-305 targeting G-protein-coupled receptor class 5 member D (GPRC5D) antibody drug conjugate for the treatment of multiple myeloma. Cancer Res. 2022;82 12_Supplement:6020-.

