# Supplementary material for: Loss of GPRC5D enhances the proliferative capacity and competitive fitness of myeloma upon anti-GPRC5D immunotherapy
Source: Leukemia. 2026 Mar 31;40(6):1227–39. doi: 10.1038/s41375-026-02920-7 (PMC13233306; doi:10.1038/s41375-026-02920-7)
Supplement: Supplementary file 1 — Supplemental material [file 41375_2026_2920_MOESM1_ESM.pdf]

# Loss of *GPRC5D* enhances the proliferative capacity and competitive fitness of myeloma upon anti-GPRC5D immunotherapy

Umair Munawar[1], Johanna Thurner[2], Silvia Nerreter[1], Thomas Nerreter[1], Alexander M. Leipold[3,4], Seungbin Han[1], Christina Verbruggen[1], Elena Gerhard-Hartmann[5], Cornelia Vogt[1], Björn Grams[1], Shilpa Kurian[1], Emma Besant[1], Sabine Roth[5], Julia Weingart[6], Patrick Eiring[6], Marietta Truger[7], Nazia Afrin[8], Torsten Steinbrunn[1,9], Yoko Tamamushi[1], Xiang Zhou[1], Nina Rein[1], Johanna Lehmann[1], Max Köppel[1], Andreas Rosenwald[5], Claudia Haferlach[7], Michael Hudecek[1], Antoine-Emmanuel Saliba[3,4], Leo Rasche[1,8], Markus Sauer[6], Hermann Einsele[1], Bernhard Kuster[2], Johannes Waldschmidt[1,10], K. Martin Kortüm[1*]

[1] Department of Internal Medicine II, University Hospital of Wuerzburg, Germany

[2] School of Life Sciences, Technical University of Munich, Freising, Germany

[3] Helmholtz Institute for RNA-based Infection Research, Helmholtz Centre for Infection Research, Wuerzburg, Germany

[4] Institute of Molecular Infection Biology, University of Wuerzburg, Würzburg, Germany.

[5] Institute of Pathology, University of Wuerzburg, Germany

[6] Department of Biotechnology and Biophysics, University of Wuerzburg, Germany

[7] MLL Munich Leukemia Laboratory, Munich, Germany

[8] Mildred Scheel Early Career Center (MSNZ), University Hospital Wuerzburg, Germany

[9] Department of Medical Oncology, Dana-Farber Cancer Institute, Boston, USA

[10] INTERACT Advanced Clinician Scientist-Program, University of Wuerzburg, Germany

# Supplementary Methods

## Cytotoxicity assay

Cells were treated with various concentrations of talquetamab in the presence of T cells from healthy donors. Three different effector-to-target-ratios (E:T) were used (1:1, 5:1, 10:1) or are otherwise mentioned in each experiment. After 48 hours of incubation with talquetamab luciferin was added, incubated at 37°C for 5 minutes before bioluminescence was measured with Tecan Infinite 200 pro plate reader (Tecan). Specific lysis was calculated using control of untreated cells without effector cells. For CAR-T cytotoxicity assays, MOCK T cells or CAR-T cells were added at different E:T ratio and were incubated for 24-72 hours before performing luciferin-based measurement. MOCK T cells were used as controls for calculating specific lysis.

## dSTORM imaging

A laser (iBeam smart 640-S Toptica, Photonics AG) was used to excite AF647. A bandpass filter (642/10, Semrock) was used for spectral cleaning of excitation light before it was focused on back focal place of the objective. The lens system and mirrors were arranged on a linear translation stage to switch between different illumination modes (EPI, HILO and TIRF illumination). The fluorescence emission was collected by the same objective and transmitted by the dichroic beam splitter (FF545/650-Di01, Semrock). A long pass filter (647 nm RazorEdge, Semrock) was used to filter emission light, and it was projected onto an electron-multiplying CCD camera (iXon DU-897D-CS0-BV, Andor Technology Ltd). Additional lenses were placed in a detection path and a final pixel size of 133nm was generated. 15,000 frames were recorded with a frame rate of ~50 Hz (20 ms exposure time) and an excitation intensity of ~3 kW/cm$^2$. A reconstructed dSTORM image and a table with all localizations were generated from recorded imaging using the single-molecule localization software rapidSTORM 3.3(1). An appropriate region of interest at the basal membrane of the cell, was

chosen using the Napari viewer implemented in the analysis toolbox LOCAN (available from: https://zenodo.org/record/5722473) was used for analysis of each *d*STORM image. The DBSCAN(2, 3) algorithm with the parameters ε = 20 nm and minimum points (MinPts) = 3 was used for clustering of localization data which were depicted as a box plot using Prism GraphPad.

**Bulk RNA-seq**

Total RNA was isolated from cell models using the RNeasy kit following the manufacturer´s instructions (Qiagen). For transcriptome analysis, 250 ng of total RNA per sample were used to prepare standard libraries (TruSeq Total Stranded RNA, Illumina). RNA was sequenced on a NovaSeq 6000 (Illumina) at 2x100 bp paired-end with a median of 64 million reads per sample. STAR, version 2.5.0a was used to align these reads to the human reference genome (hg19) and to estimate gene counts using Cufflinks (version 2.2.1). Data normalization was performed to calculate $Log_2$TPM values.

**Sample preparation for MS-based proteomics**

Cells were lysed in SDS lysis buffer (2% SDS in 40 mM Tris-HCl, pH 7.6). The samples were incubated at 95 °C for 10 min and trifluoroacetic acid (TFA) was added to a final concentration of 1%. To neutralize the sample (final pH 7.6-8.0), 20% N-methylmorpholine was added to a final concentration of 2%. Using the PierceTM BCA Protein Assay Kit (Thermo Fisher Scientific) according to the manufacturer's protocol, the protein concentration of the cell lysate was determined. The beads suspension for SP3 sample workup was prepared by mixing magnetic SeraMag-A and SeraMag-B beads (10 µl per sample of each type; Cytiva) in a ratio of 1:1, washing them three times with ddH2O and resuspending them in 10 µl dd$H_2O$ per sample. SP3 protein digestion(4) was performed on the BRAVO automatic liquid handling robot (Agilent) using a personalized protocol. 200 µg per sample was mixed with a 10 µl beads

suspension in a 96-deep well plate. Solvent-filled reservoirs and sample plates were placed onto the robot plate deck, as well as a customized magnetic plate holder. In the automated SP3 protocol, proteins were aggregated to the beads by adding ethanol to a final concentration of 70%, and 15 min incubation at 1000 rpm. The sample plate was transferred onto the magnetic plate holder for 5 min. The supernatant was removed, beads were washed with organic solvent, and after another 5 min on the magnetic plate holder the supernatant was again removed. This process was repeated three times (twice with 80% ethanol as organic solvent, and once with 100% acetonitrile (ACN)). The sample plate was removed from the robot plate deck, and beads were resuspended in 100 µl digestion buffer (100 mM HEPPS, 2 mM CaCl2, pH 8.5). Proteins were reduced with 10 mM dithiothreitol (DTT) for 40 min at 40 °C and 1200 rpm, and alkylated with 55 mM chloroacetamide (CAA) at room temperature in the dark for 20 min. Proteins were digested using trypsin (1:50 trypsin-to-protein ratio, Roche) at 37 °C and 1000 rpm overnight. Peptide recovery was performed the next day using BRAVO by settling the beads onto the magnet, and by transfer of the supernatant to a sample collection plate. Beads were washed once with 120 µl 2% TFA to recover remaining peptides and acidify the digest. The supernatant was added to the sample collection plate. Peptides clean-up was performed by desalting on SPE MULTI 96-Monoblock CHROMABOND HLB plates (96x 10 mg, 30 µm, Macherey-Nagel). The desalting plates were washed with isopropanol, ACN and 70% ACN/0.1% TFA (centrifugation at 200 rpm, 5 min each). Plates were equilibrated using 0.1% TFA, and samples were loaded by gravity. Flow-through was re-loaded, samples were washed with 0.1% TFA, and peptides were eluted with 70% ACN/0.1% TFA. Samples were frozen at -80 °C and dried in a SpeedVac. Labelling of the desalted peptides (200 µg protein per channel) was performed using TMTpro 18-plex (Thermo Fisher Scientific) by dissolving peptides in 20 µl 200 mM 3-[4-(2-Hydroxyethyl)piperazin-1-yl]propane-1-sulfonic acid buffer (HEPPS, pH 8.5). For each treatment condition and replicate, one TMT channel was used

(126/127N/127C: GPRC5D$^{WT}$ + Talquetamab R1-3; 128N/128C/129N: GPRC5D$^{WT}$ R1-3; 129C/130N/130C: GPRC5D$^{WT/Del}$ + Talquetamab R1-3; 131N/131C/132N: GPRC5D$^{WT/Del}$ R1-3; 132C/133N/133C: GPRC5D$^{Del/Del}$ + Talquetamab R1-3; 134N/ 134C/135N: GPRC5D$^{Del/Del}$ R1-3). The dry TMT reagent was thawed, dissolved in 16 µl anhydrous ACN, and immediately used. 5 µl TMT reagent (156 µg) was added per sample and the samples were incubated for 1 hour at 20 °C and 400 rpm while the labelling reaction occurred. The reaction was stopped by adding 2 µl 5% hydroxylamine per sample and incubated for 15 min at 20 °C and 400 rpm. Afterwards, all samples were combined, acidified to 1% formic acid (FA), and dried using a SpeedVac. The sample was reconstituted in 0.1% FA and desalted using 50 mg tC18 RP cartridges (Waters) in a vacuum manifold (wash solvent: 0.1% FA, elution solvent: 0.1% FA in 60% ACN). A Vanquish HPLC (Thermo Fisher Scientific) system operating a Waters XBridge BEH130 4.6 x 250 mm C18 reverse-phase column was used to fractionate the pooled peptides (reconstituted in 25 mM ammonium bicarbonate, pH 8.0), at a 1 ml/min flow rate and column oven temperature of 45 °C. Peptides were separated by linear gradients from 1% to 7% ACN in 4 min, followed by 7% to 45 % ACN in 42 min, and from 45% to 80% ACN in 7 min. The proportion of solvent A (25 mM ammonium bicarbonate, pH 8.0) was kept at 10% during separation. Fractions were collected every 30 seconds, starting from minute 7 of the gradient, amounting to a total of 96 fractions. Each fraction was acidified to 1% FA.

Phospho-enrichment was performed with the remaining sample. Fractionated samples were dissolved in 0.1% FA/60% ACN and pooled from 96 into 12 fractions (fraction 1, 49, 25 and 13, fraction 2, 50, 25 and 14, and so on). Phospho-enrichment was done using Fe(III)-NTA 5 µl IMAC cartridges on the Agilent BRAVO automatic liquid handling robot and the standard protocol for phosphopeptides enrichment 2.0. Cartridges were primed using 150 µl 0.1% TFA/ACN and equilibrated with 150 µl 80% ACN/0.1%TFA. Sample was loaded onto the cartridges and washed using 150 µl 80% ACN/0.1% TFA. Bound peptides were eluted with

60 µl 1% ammonium hydroxide. Eluted phosphopeptides were dried down and used for LC-MS analysis.

**LC-MS3 analysis**

Samples were measured on a Orbitrap Eclipse Tribrid mass spectrometer (Thermo Fisher Scientific) coupled to an Ultimate 3000 RSL cnano system (Thermo Fisher Scientific). Phosphoproteome samples were reconstituted in 0.1% FA, and peptides were separated using an in-house packed C18 trap column (75 µm x 2 cm, 5 µm Reprosil resin; Dr. Maisch) and an in-house packed Reprosil C18 analytical column (75 µm x 48 cm, 1.9 µm resin; Dr. Maisch) with a flow-rate of 300 nl/min. The following solvents were used: A: 0.1% FA, 5% DMSO in ddH2O; B: 0.1% FA, 5% DMSO in ACN. A two-step 90 min linear gradient was used: 4% to 22.5% to 32% of solvent B in solvent A. The mass spectrometer was operated in positive ionization and DDA mode, with a spray voltage of 2 kV. MS1 spectra were recorded in the orbitrap with a scan range of 360-1,500 m/z and resolution set to 60,000. The AGC target value was set to 4e5 with a maximum IT of 50 ms and RF lens value set to 50%. MIPS mode was set as peptide and default charges were set to 2-6. The dynamic exclusion duration was 90s, with exclude after 1 time. For readout of MS2 spectra, the orbitrap was used at 30,000 resolution. Isolation window was set to 0.7 m/z and precursor ions were fragmented using CID with 32% collision energy. The AGC target value was set to 5e4 at a maximum IT of 60 ms and spectra were recorded in a mass range from 400-2,000 m/z including a neutral loss mass of 97.9769 m/z. For each MS2 an additional MS3 spectrum (scan range 100-1,000 m/z, 2.5e5 AGC target, maxIT 120 ms) was acquired after HCD fragmentation with 55% collision energy and synchronous precursor selection at 50k resolution in the orbitrap.

**Peptide and protein identification and quantification**

Peptide and protein identification and quantification was performed using MaxQuant(5) (version 2.6.6.0) by searching the MS raw files against all canonical protein sequences as annotated in the UniProt reference database (reviewed human proteins only, 20412 entries, downloaded 20$^{th}$ of November 2024) using the search engine Andromeda(6). Carbamidomethylated cysteine was set as a fixed modification, oxidation of methionine, N-terminal protein acetylation, and phosphorylation of serine, threonine, and tyrosine (phospho(STY)) were set as variable modifications. The proteolytic enzyme was specified as Trypsin/P, and up to two missed cleavage sites were allowed. MS3-based TMT quantification was enabled, taking TMT correction factors as supplied by the manufacturer into account. All other search parameters were left as the default suggested by MaxQuant.

**scRNA-seq sample preparation**

For multiplexed single-cell experiments, cell hashing was performed using TotalSeq-C hashtag antibodies and the TotalSeq Universal Cocktail (BioLegend). Briefly, each sample was stained with a unique TotalSeq-C hashtag antibody in combination with the universal cocktail to enable both sample multiplexing and quantification of cell surface proteins. Staining was carried out according to the manufacturer´s recommended protocol. In brief, cell suspensions were incubated with antibodies for 30 minutes on ice, followed by three washes in cold staining buffer to remove unbound antibodies. After staining, samples were pooled in equal cell numbers and counted prior to encapsulation.

Pooled cells were then loaded into the 10x Genomics Chromium X controller using the 5' GEM-X gene expression workflow. Encapsulation into Gel Beads-emulsion (GEMs) was carried out following the manufacturer’s instructions. Within each GEM, cells were lysed and mRNAs, as well as hashtag oligo (HTO) sequences, were captured by barcoded oligonucleotides present on the beads. Each cDNA molecule was labeled with a cell-specific

barcode and unique molecular identifier (UMI) during reverse transcription, allowing for transcript quantification at the single-cell level and identification of the sample of origin. Following reverse transcription, emulsions were broken, and cDNA was recovered. Library preparation proceeded with the 10x Genomics 5' v3 chemistry. Final libraries comprising GEX and HTO-derived hashing libraries were quantified, pooled and sequenced on an Illumina NovaSeq X plus platform.

**scRNA-seq data analysis**

Samples were demultiplexed based on centered log-ratio (CLR) normalized hashtag oligo (HTO) counts using the HTODemux function (default parameters). Only cells flagged as singlets were retained for further analysis. Additionally, cells with more than 10% mitochondrial count fraction were filtered out as low-quality cells. Gene expression counts were log-normalized, highly variable features were computed and scaled. Principal component analysis (PCA) was performed on scaled highly variable features. ADT counts were CLR normalized, scaled and subjected to PCA. Weighted Nearest Neighbor (WNN) analysis was used to integrate modalities by constructing a joint neighbor graph. The WNN graph was used to compute a two-dimensional embedding by Uniform Manifold Approximation and Projection (UMAP), followed by unsupervised clustering using the Louvain algorithm. MM cell clusters were identified based on expression of known marker genes, and labeled according to their condition and genetic background. T cell clusters were annotated based on known marker genes. Differential gene expression was performed using the Seurat implementation of the Wilcoxon rank-sum test in the function FindMarkers. Scores for interferon response signatures were computed using the AddModuleScore function with default parameters. Enrichment analysis was performed using GSEA (v4.4.0).

**Clonal competition assay**

For each technical replicate, 1x10e6 cells were seeded in T25 cell culture flasks under standard cell culture conditions. Sequential samples were taken for flow cytometry analysis on regular intervals, and cell cultures were split every 3-4 days. Cells were washed with PBS twice before resuspending in FACS buffer. A total of 20,000 cells were counted for CytoFLEX (Beckman Coulter) and relative ratio/numbers were calculated using FlowJo version 10.1.

**Vector construction**

epHIV7 lentiviral vectors containing CARs were constructed as previously described(7). Briefly, cDNA encoding scFvs targeting GPRC5D (Talq and MCARH109) were synthesized (Thermo Fisher Scientific) and subcloned into existing lentiviral backbones to yield 2nd generation CARs containing an IgG4-derived Spacer, a CD28 transmembrane domain, and a 4-1BB (CD137) costimulatory domain followed by a CD3zeta (CD247) signaling domain. A truncated epidermal growth factor (EGFRt) separated from the CAR transgene via a T2A ribosomal skip element sequence(8, 9) was included in all vectors to allow assessment of CAR-positivity and enrichment of $CAR^+$ T cells.

**Preparation of lentivirus for transduction**

Lentiviral supernatants were generated using Lenti-X 293T cells (Takara), as previously described(10). Briefly, Lenti-X 293T cells were seeded in 10mm Petri dishes and co-transfected with the respective lentiviral vector plasmids and the packaging vectors pCHGP-2, pCMV-Rev2, and pCMV-G using Calphos transfection reagent (Takara). The culture medium was changed 16 hours after transfection, and lentiviral supernatants were collected after 72 hours. Lentiviral particles were purified via density gradient ultracentrifugation performed at 24,900 rpm for 2 hours at 4 ºC, and stored at -80°C until use.

**CAR-T cell generation**

Human CART cells were generated as previously described(7, 11). In brief, human PBMCs were isolated by density gradient centrifugation using Pancoll (PAN-Biotech). CD4+ and CD8+ T cells were obtained by negative immunomagnetic isolation (CD4+ and CD8+ T Cell Isolation Kit, human, Miltenyi Biotech) and subsequently activated with anti-CD3/CD28 Dynabeads (Miltenyi Biotech) at a bead-to-cell ratio of 1:1. The activated T cells were transduced on day 1 after activation by spinoculation with lentiviral supernatants at an MOI of 3. The transduced T cells were maintained in T cell medium consisting of RPMI-1640 medium (Thermo Fisher Scientific) supplemented with 1% GlutaMAX (Thermo Fischer Scientific), 100 u/ml penicillin/streptomycin, 10% heat-inactivated human serum, 50 μM 2-Mercaptoethanol (Sigma Aldrich) and 50 u/ml rhIL-2 (Miltenyi Biotech). Anti-CD3/CD28 Dynabeads were removed on day 6 after T cell isolation, and CAR$^+$ T cells were enriched via antibody-mediated magnetic sorting using the EGFRt marker. Subsequently, T cells were further expanded by polyclonal stimulation as described(12). In brief, generated CAR-T cells were co-cultivated with irradiated allogeneic PBMCs, TM-LCL, and 30 ng/ml anti-CD3 mAb (Okt-3) (Miltenyi Biotech) in T cell medium supplemented with 50 u/ml rhIL-2 (Miltenyi Biotech). T cells were used for functional assays *in vitro* between day 1 and 22 after expansion.

**Cytokine profiling by Sciomics**

The samples were labelled at an adjusted protein concentration for two hours with scioDye 2 (Sciomics). The reference sample was labelled with scioDye 1 (Sciomics). After two hours the reaction was stopped and the buffer exchanged to PBS. All labelled protein samples were stored at -20° C until use. The 12 samples were analyzed in a dual-color approach using a reference based design on 12 scioCyto antibody microarrays (Sciomics) targeting a variety of cytokines and chemokines. Each antibody is represented on the array in four replicates. The arrays were blocked with scioBlock (Sciomics) on a Hybstation 4800 (Tecan, Austria) and afterwards the samples were incubated competitively with the reference sample using a dual-color approach.

After incubation for three hours, the slides were thoroughly washed with 1x PBSTT, rinsed with 0.1x PBS as well as with water and subsequently dried with nitrogen(13).

**Data acquisition and analysis**

Slide scanning was conducted using a Powerscanner (Tecan, Austria) with constant instrument laser power and PMT settings. Spot segmentation was performed with GenePix Pro 6.0 (Molecular Devices, Union City, CA, USA). Acquired raw data were analyzed using the linear models for microarray data (LIMMA) package of R-Bioconductor after uploading the median signal intensities(14). For normalization, a specialized invariant Lowess method was applied(15). For analysis of the samples, a one-factorial linear model was fitted via least squares regression with LIMMA, resulting in a two-sided t-test or F-test based on moderated statistics. All presented p values were adjusted for multiple testing by controlling the false discovery rate according to Benjamini and Hochberg(16).

Differences in protein abundance between different samples or sample groups are presented as log-fold changes (logFC) calculated for the basis 2. In a study comparing samples versus control a logFC = 1 means that the sample group had on average a $2^1 = 2$ fold higher signal than the control group. logFC = -1 stands for $2^{-1} = 1/2$ of the signal in the sample as compared to the control group. Proteins with a logFC > 0.5 and an adjusted p value < 0.05 were defined as differential and displayed in blue in the following volcano plots.

# Supplementary Results

## Figure S1

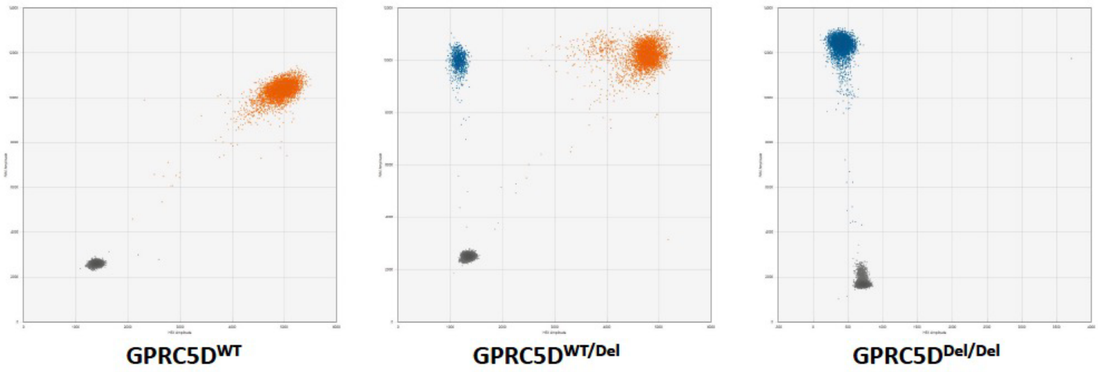


**Figure S1. Two-dimensional droplet fluorescence intensity plots of NHEJ drop-off assay.** Droplets with WT cluster being positive for both FAM and HEX represented as orange cluster on top right side. Droplets with NHEJ mutation positive cluster are positive for FAM only and represented in blue on top left side.

# Figure S2

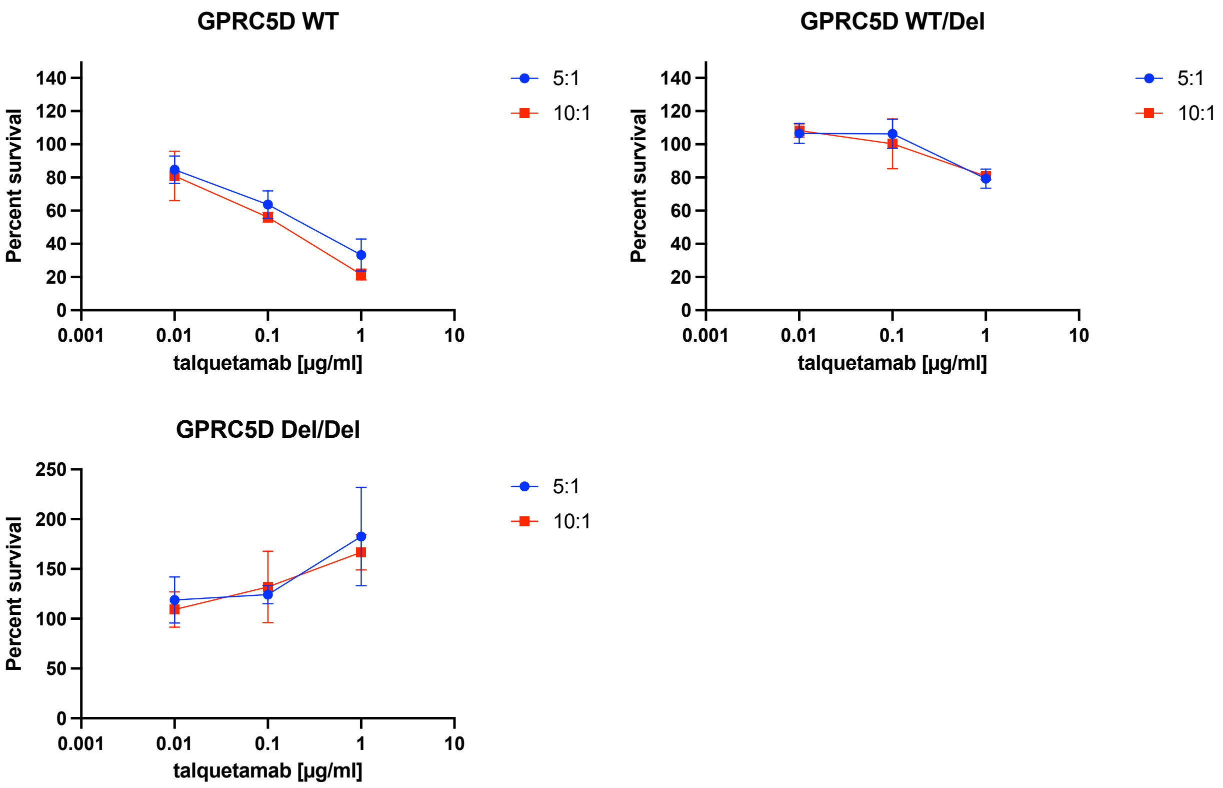


**Figure S2. Talquetamab cytotoxicity assay performed with different E:T cell ratios.** Bioluminescence-based cell survival analysis performed on GPRC5D models treated with various concentrations of talquetamab and two different E:T cells ratio for 48 hours. *E:T ratio of 5:1* is represented in Blue, *E:T ratio of 10:1* is represented in red.

Figure S3

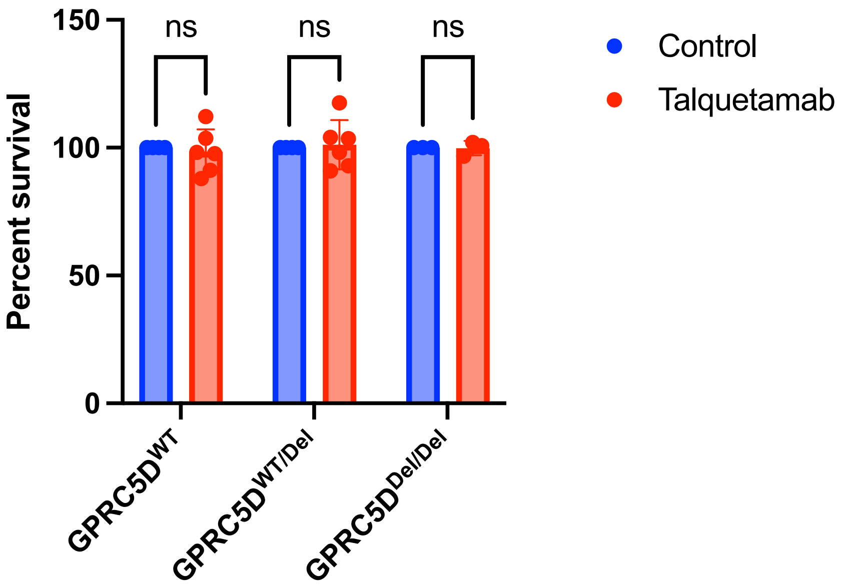


**Figure S3. Impact of Talquetamab treatment in absence of effector cells.** GPRC5D and deficient cells models were treated with 10µg/ml talquetamab or PBS (Solvent Control) for 48 hours and luciferin-based cytotoxicity assay was performed.

# Figure S4

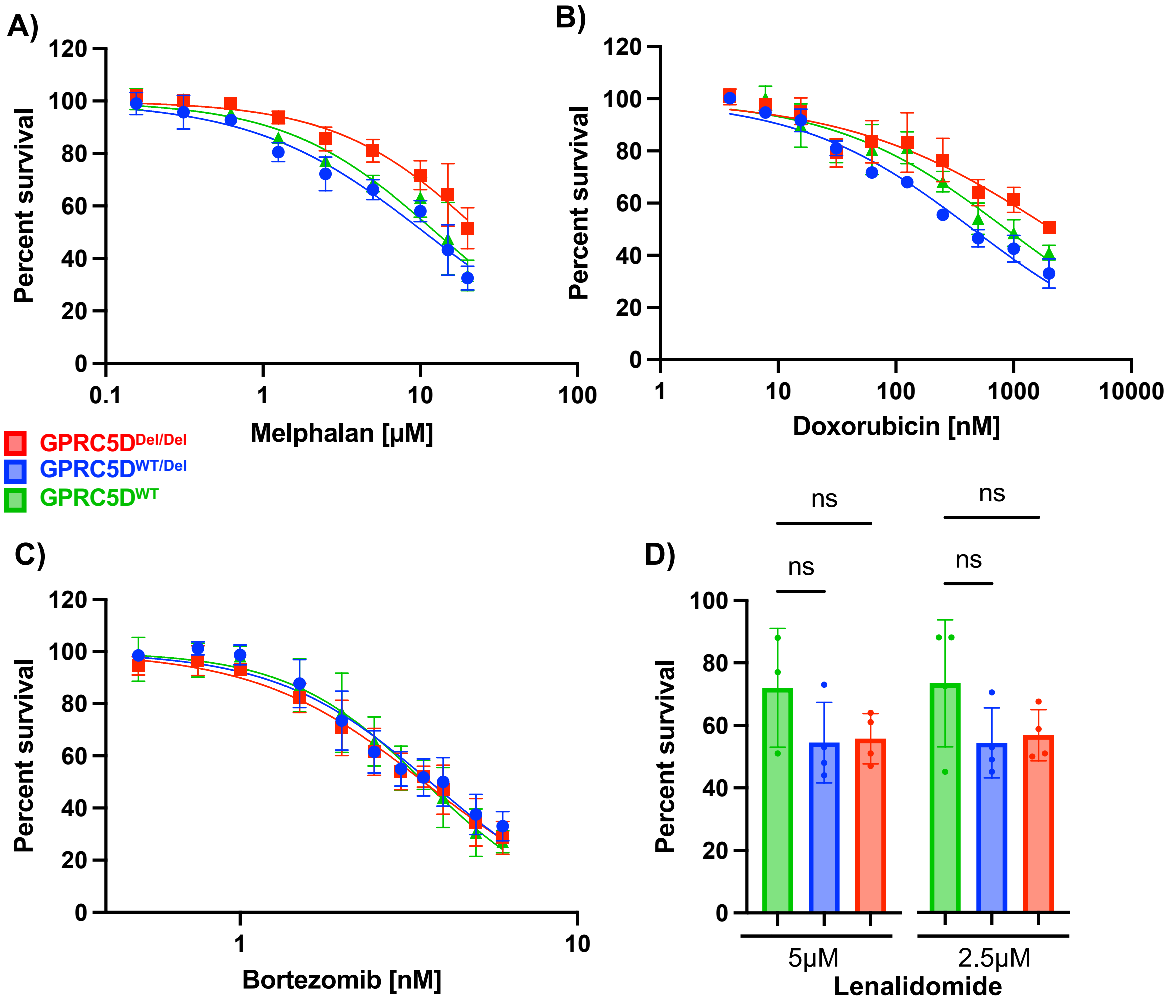


**Figure S4. Sensitivity of GPRC5D cell line models towards conventional antimyeloma agents.** Cells were treated with increasing does of melphalan (A), doxorubicin (B) and bortezomib (C) for 72 hours, and with lenalidomide for 120 hours (D). Cytotoxicity was measured using the AlamarBlue assay.

# Figure S5

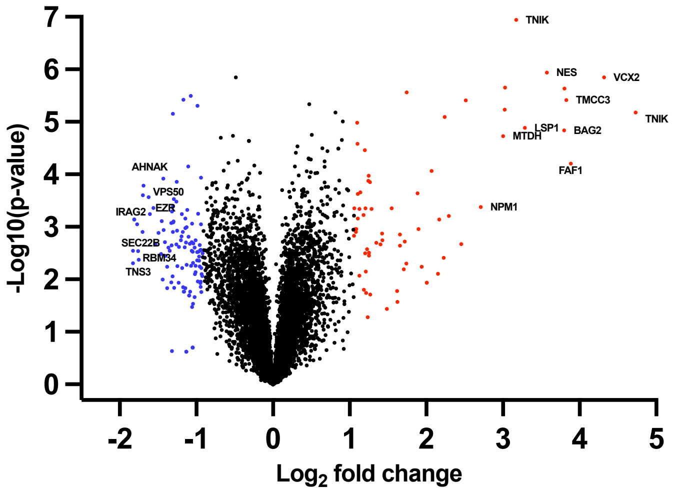


**Figure S5. Phosphoproteomic landscape in response to talquetamab treatment.** Volcano plot showing differentially expressed phosphosites between talquetamab treated GPRC5D$^{Del/Del}$ and GPRC5D$^{WT}$ models at a log 2FC > │1│change.

Figure S6

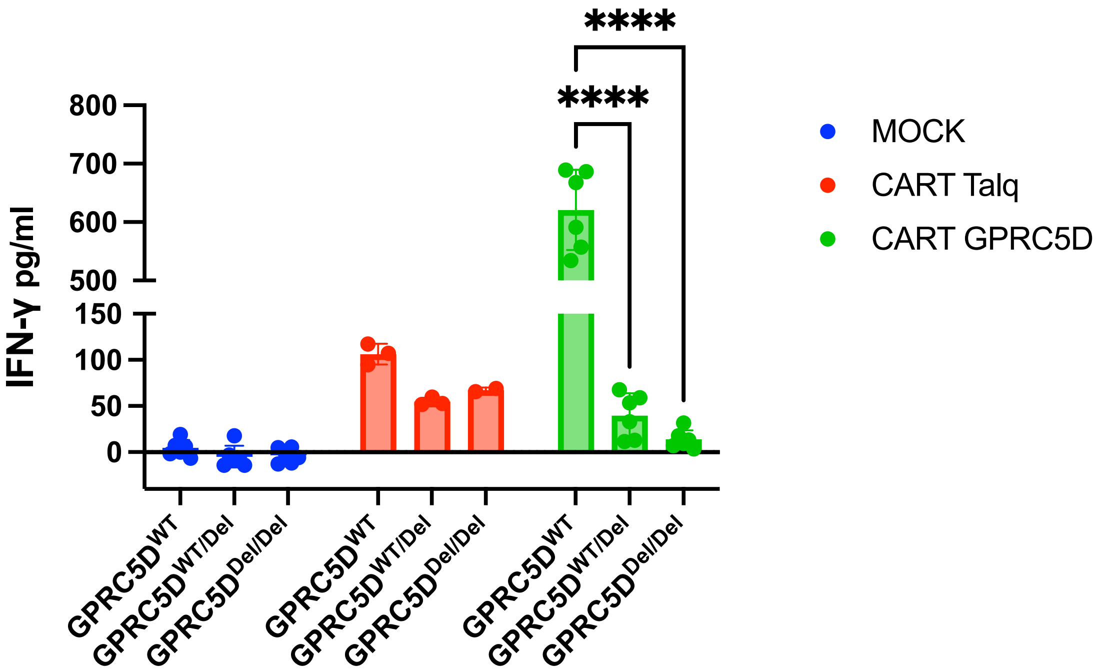


**Figure S6. IFN-γ quantification for functional profiling of CAR-T cells in the presence vs. absence of GPRC5D. A)** Bar graphs representing the levels of IFN-γ in the supernatant. GPRC5D cell models (WT and knock-out) were treated with either CART-Talq or CART-GPRC5D cells for 24 hours and the supernatants were analyzed for IFN-γ production.

Figure S7

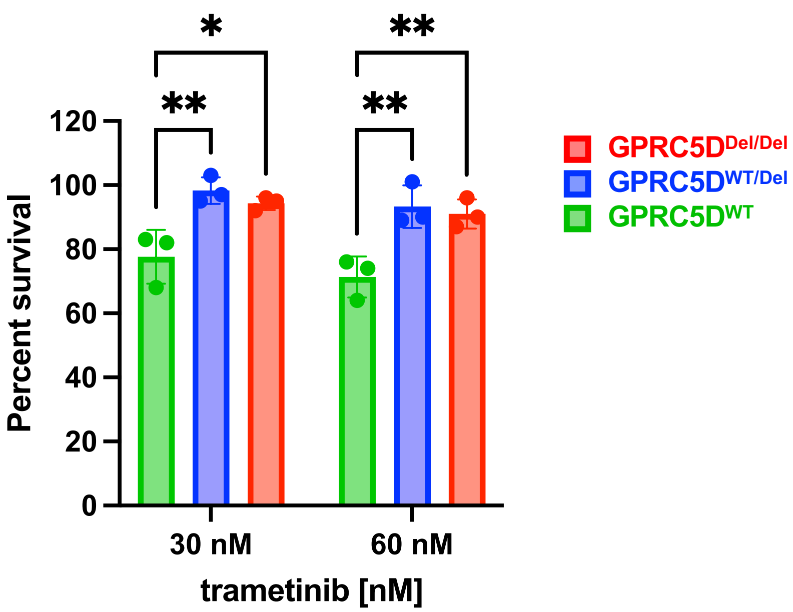


**Figure S7. Sensitivity of GPRC5D cell line models towards MEK inhibitor trametinib.** Cells were treated with 30 and 60nM trametinib for 72 hours. Cytotoxicity was measured using the AlamarBlue assay. *p<0.05, **p<0.005

# Figure S8

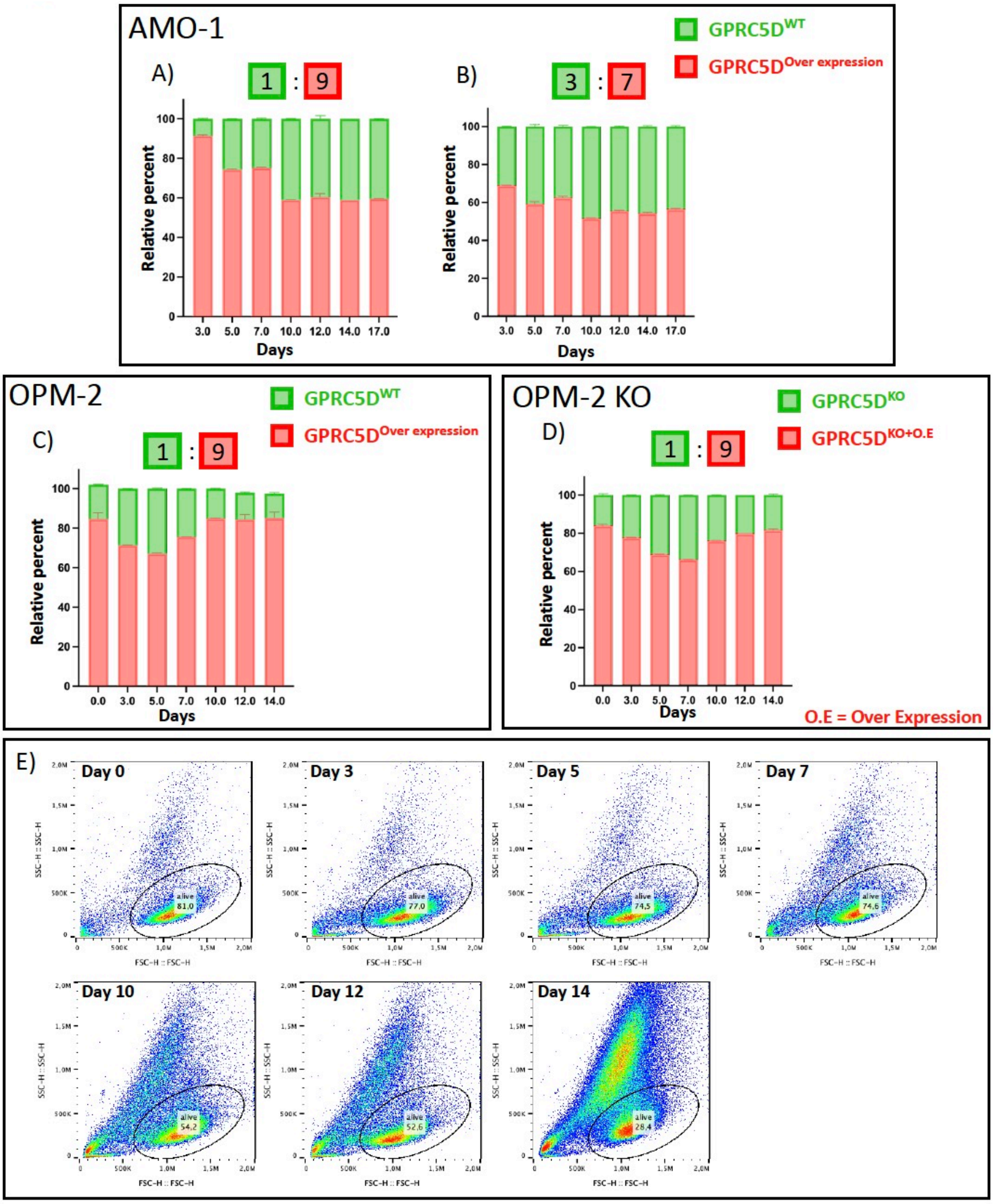


**Figure S8. Functional impact of GPRC5D over expression.** Clonal competition assay showing the selection dynamics of co-cultures AMO-1 cell line **(A,B)** OPM-2$^{WT}$ **(C)** and OPM-2$^{KO}$ **(D)** cell models in various starting ratios of GPRC5D over expressing cells and respective

native cell models. **(E)** Representative flow images showing overall health of the co-culture over time course of co-culture experiment.

## References


1. Wolter S, Löschberger A, Holm T, Aufmkolk S, Dabauvalle M-C, Van De Linde S, et al. rapi d STORM: accurate, fast open-source software for localization microscopy. Nature methods. 2012;9(11):1040-1.
2. Ester M, Kriegel H-P, Sander J, Xu X, editors. A density-based algorithm for discovering clusters in large spatial databases with noise. kdd; 1996.
3. Ebert V, Eiring P, Helmerich DA, Seifert R, Sauer M, Doose S. Convex hull as diagnostic tool in single-molecule localization microscopy. Bioinformatics. 2022;38(24):5421-9.
4. Hughes CS, Moggridge S, Müller T, Sorensen PH, Morin GB, Krijgsveld J. Single-pot, solid-phase-enhanced sample preparation for proteomics experiments. Nature protocols. 2019;14(1):68-85.
5. Cox J, Mann M. MaxQuant enables high peptide identification rates, individualized p.p.b.-range mass accuracies and proteome-wide protein quantification. Nat Biotechnol. 2008;26(12):1367-72.
6. Cox J, Neuhauser N, Michalski A, Scheltema RA, Olsen JV, Mann M. Andromeda: a peptide search engine integrated into the MaxQuant environment. J Proteome Res. 2011;10(4):1794-805.
7. Hudecek M, Sommermeyer D, Kosasih PL, Silva-Benedict A, Liu L, Rader C, et al. The nonsignaling extracellular spacer domain of chimeric antigen receptors is decisive for in vivo antitumor activity. Cancer immunology research. 2015;3(2):125-35.

8. Paszkiewicz PJ, Fräßle SP, Srivastava S, Sommermeyer D, Hudecek M, Drexler I, et al. Targeted antibody-mediated depletion of murine CD19 CAR T cells permanently reverses B cell aplasia. The Journal of clinical investigation. 2016;126(11):4262-72.

9. Wang X, Chang W-C, Wong CW, Colcher D, Sherman M, Ostberg JR, et al. A transgene-encoded cell surface polypeptide for selection, in vivo tracking, and ablation of engineered cells. Blood, The Journal of the American Society of Hematology. 2011;118(5):1255-63.

10. Mestermann K, Giavridis T, Weber J, Rydzek J, Frenz S, Nerreter T, et al. The tyrosine kinase inhibitor dasatinib acts as a pharmacologic on/off switch for CAR T cells. Science translational medicine. 2019;11(499):eaau5907.

11. Feucht J, Sun J, Eyquem J, Ho Y-J, Zhao Z, Leibold J, et al. Calibration of CAR activation potential directs alternative T cell fates and therapeutic potency. Nature medicine. 2019;25(1):82-8.

12. Riddell SR, Greenberg PD. The use of anti-CD3 and anti-CD28 monoclonal antibodies to clone and expand human antigen-specific T cells. Journal of immunological methods. 1990;128(2):189-201.

13. Schröder C, Jacob A, Tonack S, Radon TP, Sill M, Zucknick M, et al. Dual-color proteomic profiling of complex samples with a microarray of 810 cancer-related antibodies. Mol Cell Proteomics. 2010;9(6):1271-80.

14. Ritchie ME, Phipson B, Wu D, Hu Y, Law CW, Shi W, et al. limma powers differential expression analyses for RNA-sequencing and microarray studies. Nucleic Acids Res. 2015;43(7):e47.

15. Sill M, Schröder C, Hoheisel JD, Benner A, Zucknick M. Assessment and optimisation of normalisation methods for dual-colour antibody microarrays. BMC Bioinformatics. 2010;11:556.

16. Benjamini Y, Hochberg Y. Controlling the False Discovery Rate: A Practical and Powerful Approach to Multiple Testing. Journal of the Royal Statistical Society: Series B (Methodological). 1995;57(1):289-300.
